# *Streptococcus pneumoniae*: a Plethora of Temperate Bacteriophages With a Role in Host Genome Rearrangement

**DOI:** 10.3389/fcimb.2021.775402

**Published:** 2021-11-18

**Authors:** Antonio J. Martín-Galiano, Ernesto García

**Affiliations:** ^1^ Intrahospital Infections Laboratory, National Centre for Microbiology, Instituto de Salud Carlos III (ISCIII), Majadahonda, Spain; ^2^ Departamento de Biotecnología Microbiana y de Plantas, Centro de Investigaciones Biológicas Margarita Salas (CSIC), Madrid, Spain; ^3^ Centro de Investigación Biomédica en Red de Enfermedades Respiratorias (CIBERES), Madrid, Spain

**Keywords:** *Streptococcus pneumoniae*, prophage, integrase, endolysin, lytic enzymes, tRNAs, virulence factors, genomic rearrangements

## Abstract

Bacteriophages (phages) are viruses that infect bacteria. They are the most abundant biological entity on Earth (current estimates suggest there to be perhaps 10^31^ particles) and are found nearly everywhere. Temperate phages can integrate into the chromosome of their host, and prophages have been found in abundance in sequenced bacterial genomes. Prophages may modulate the virulence of their host in different ways, e.g., by the secretion of phage-encoded toxins or by mediating bacterial infectivity. Some 70% of *Streptococcus pneumoniae* (the pneumococcus)—a frequent cause of otitis media, pneumonia, bacteremia and meningitis—isolates harbor one or more prophages. In the present study, over 4000 *S. pneumoniae* genomes were examined for the presence of prophages, and nearly 90% were found to contain at least one prophage, either defective (47%) or present in full (43%). More than 7000 complete putative integrases, either of the tyrosine (6243) or serine (957) families, and 1210 full-sized endolysins (among them 1180 enzymes corresponding to 318 amino acid-long *N*-acetylmuramoyl-L-alanine amidases [LytA_PPH_]) were found. Based on their integration site, 26 different pneumococcal prophage groups were documented. Prophages coding for tRNAs, putative virulence factors and different methyltransferases were also detected. The members of one group of diverse prophages (PPH090) were found to integrate into the 3’ end of the host *lytA_Spn_* gene encoding the major *S. pneumoniae* autolysin without disrupting it. The great similarity of the *lytA_Spn_* and *lytA*_PPH_ genes (85–92% identity) allowed them to recombine, *via* an apparent integrase-independent mechanism, to produce different DNA rearrangements within the pneumococcal chromosome. This study provides a complete dataset that can be used to further analyze pneumococcal prophages, their evolutionary relationships, and their role in the pathogenesis of pneumococcal disease.

## Introduction

Bacteriophages (phages) are viruses that infect bacteria. They are the most abundant biological entities on Earth — current estimates suggest there to be close to 10^31^ phage particles (Mushegian, 2020) — and can be found nearly everywhere. Temperate bacteriophages infect and kill bacteria to release phage progeny, but on occasion they may integrate into the host genome *via* site‐specific recombination events, and replicate vertically. Integrated phages are stably maintained in the chromosome (i.e., as a prophage) in a state known as lysogeny (Lwoff, 1953). Campbell (1962) was the first to propose a model for the integration of λ prophage into the bacterial chromosome. This model consists of two phases: (1) the circularization of the linear phage DNA molecule injected into the cell, and (2) the linear insertion of the phage DNA into the bacterial chromosome *via* the activity of a specific integrase (Int) that catalyzes the site-specific recombination of the phage attachment site (*attP*) and bacterial attachment site (*attB*). These two sequences generally share a short stretch of identical bases (the core sequence) where this site-specific recombination occurs. After recombination, the phage genome is left integrated into the bacterial chromosome, flanked by two duplicated hybrid *att* sites: *attL* and *attR*. Under certain circumstances (prophage induction), the prophage becomes excised from the bacterial chromosome and viral replication begins *via* the lytic cycle.

Endolysins are phage-encoded enzymes capable of hydrolyzing the bacterial cell wall; they are synthesized at the end of the lytic cycle to allow the release of phage progeny. In this era of global increase in antibacterial resistance, endolysins are being tested as an alternative (or complement) to the use of antibiotics (for recent reviews see Vázquez et al., 2018; Fernández et al., 2021; Murray et al., 2021). It is well documented that, while integrated into the host genome, genes encoding Ints and endolysins are located at either end of *Streptococcus* prophages (Canchaya et al., 2003).

Prophages are often found in sequenced bacterial genomes. Indeed, nearly half of bacterial genomes appear to contain at least one prophage. The minimum doubling time is the trait most strongly correlated with lysogeny, followed by genome size and, interestingly, pathogenicity (Touchon et al., 2016).

*Streptococcus pneumoniae* (the pneumococcus) is a major human pathogen and a frequent cause of non-invasive diseases such as otitis, conjunctivitis and pneumonia, but also life-threatening invasive sepsis, bacteremic pneumonia, and meningitis. Pneumococcal pneumonia ranks first in terms of associated mortality among all lower respiratory tract diseases, and is responsible for more than one million deaths every year (GBD 2016 Lower Respiratory Infections Collaborators, 2018). That prophages may contribute to bacterial virulence is well established, particularly in *Pseudomonas aeruginosa, Salmonella enterica, Escherichia coli, Vibrio cholerae, Staphylococcus* spp., and *Clostridium* spp. (Schroven et al., 2021). Prophages can alter the phenotype of their hosts at different levels, e.g., by causing them to secrete toxins, by modifying the bacterial envelope, and/or impacting bacterial infectivity and bacterial cell regulation.

High-throughput next generation sequencing techniques have now provided numerous *de novo* sequenced and assembled bacterial genomes, and over 8000 pneumococcal genomes are currently included in the NCBI Reference Sequence Database (RefSeq) database. However, while first reported in 1977 and suggested to be widespread (Bernheimer, 1977; Ramirez et al., 1999), pneumococcal prophages (PPHs) have remained relatively unexamined — that is until recently (García et al., 2005). Based on comparisons of complete prophages and their predicted encoded proteins, PPHs have been grouped into three major groups with only a small number of prophages falling outside these classes (Romero et al., 2009a; Romero et al., 2009b). More recently, pairwise comparisons of prophage sequences showed four major prophage clusters and one single prophage (Brueggemann et al., 2017). Phages belonging to the same phylogenetic group share high sequence similarity in their packaging, morphology, and lysis modules, and are typically associated with one or two main integrase types. Besides, incomplete pneumococcal prophages can be highly conserved over long periods of time and clustered into five major groups that differed from those of intact PPHs (Rezaei Javan et al., 2019).

In 2017, a pan-genome-wide association study identified PPHs as being associated with reduced pneumococcal carriage duration, although this was attributed more to the disruption caused by the integration of the phage genome and the genetic transformation competence system than to any property of the prophage itself (Lees et al., 2017). Other data have shown that a role for prophages in pneumococcal virulence and patient mortality is linked to PblA and/or PblB, two prophage-encoded proteins known to be involved in enhanced platelet activation together with higher formation of platelet-monocyte complexes (Tunjungputri et al., 2017). Prophages may naturally enter the lytic cycle, or be induced to do so *via* exposure to fluoroquinolones (López et al., 2014) (with a concomitant increase in PblA/PblB expression). A recent study involving patients with invasive pneumococcal disease determined that the 30-day mortality of pneumococcal meningitis was 11% in *pblB*-positive patients compared to <1% in *pblB*-negative patients, although the authors recognize that this finding does not prove causality (Cremers et al., 2019). It has also recently been suggested that incomplete PPHs and certain prophage genes may be involved in pneumococcal pathogenesis (Rezaei Javan et al., 2019). In addition, transcriptomic analyses have shown that a defective PPH may serve as a switch that controls the expression of a bacterial gene (*ychF*) located immediately downstream of the prophage *int* gene that is involved in nasopharyngeal colonization (Chen et al., 2019). A review is available on the recent advances in the genomic and functional characterization of pneumococcal temperate phages, and their contribution to pneumococcal pathogenesis and genome evolution (Garriss and Henriques-Normark, 2020).

In the present study, the genomic sequences of over 4000 *S. pneumoniae* isolates from diverse lineages were examined to obtain the broadest possible picture of PPH diversity. The precise integration sites and the *att* core sequences of most of them were elucidated and used to identify the families to which a number of previously reported but incompletely studied PPHs belong. In addition, recombination between prophage and host *lytA* genes is shown to be an important source of chromosomal rearrangement in *S. pneumoniae*.

## Materials and Methods

### Compilation of the Pneumococcal Genome Dataset

This study was performed using a dataset mined from the National Center for Biotechnology Information (NCBI) database (available at https://www.ncbi.nlm.nih.gov/genome/?term=Streptococcus+pneumoniae). The latter contains whole-genome sequences (assembled or otherwise) for more than 8500 pneumococcal genomes (last accessed January 30, 2021). Sequence types (STs) (Enright and Spratt, 1998; Jolley et al., 2018) of the strains included in the dataset were determined on the basis of whole genome sequencing data (Larsen et al., 2012).

### Search for Pneumococcal Prophages

To detect PPHs, the dataset was searched, using the BLAST platform (Johnson et al., 2008), for homologs of *int*- and *lytA*_PPH_-like genes encoding (respectively) Ints and endolysins [with respect to the latter, only *N*-acetylmuramoyl-L-alanine amidases (NAM-amidases; EC 3.5.1.28) of the *Amidase_2* family (Pfam database identifier: PF01510) (Morales et al., 2010) are currently known]. The corresponding genes/proteins of previously characterized PPHs, such as MM1 (Obregón et al., 2003a), фSpn_OXC, фSpn_6, and фSpn_18 (Romero et al., 2009a), and others (Càmara et al., 2018). The endolysins encoded by various virulent pneumococcal phages (see below) were used as query sequences. The original and newly found genes/proteins with a coverage ≥ 90% and ≥ 50% identity were used in searches in an iterative manner.

### Bioinformatic Analyses

Sequence comparison and alignments were performed using the BLAST platform and/or Clustal Omega package (Sievers and Higgins, 2021) running at the European Bioinformatics Institute (EMBL-EBI) website (https://www.ebi.ac.uk). Potential tRNAs genes were identified using ARAGORN (Laslett and Canback, 2004) and tRNAscan-SE (Schattner et al., 2005) software. Detections were recorded only when both programs coincided in the results returned. The codon usage of *S. pneumoniae* D39 was obtained from the corresponding database (Nakamura et al., 2000). Protein domains were preliminarily identified using CDD/SPARCLE (Lu et al., 2020). PHASTER (running at https://phaster.ca/) was also employed to identify phage-homologous regions in bacterial genomes (Arndt et al., 2016). To ascertain equivalent detection of protein datasets, open reading frames were identified and translated from prophage sequences using Prodigal v2.6.3 (Hyatt et al., 2010). Prophage proteomes were then compared all against all through the calculation of the weighted gene repertoire relatedness (wGRR) (Pfeifer et al., 2021). Best bi-directional hits were found from hits detected by the ‘easy-search’ workflow of MMseqs2 (Mirdita et al., 2019) with e-value < 10^−4^, identity ≥ 35%, coverage ≥ 50% thresholds. Agglomerative hierarchical clustering from the resulting wGRR matrix was carried out by the *linkage* tool of the scipy.cluster.hierarchy python library using the ‘ward’ method on euclidean distances.

## Results

### Temperate Bacteriophages Are Abundant in *S. pneumoniae*

The dataset produced contained 4003 strains, including 126 whose genomes were sequenced to complete or near-complete (chromosome) assembly level and deposited in the RefSeq database (O'Leary et al., 2016) (last accessed, October 11, 2020) (labeled in green in **Table S1**). The strains included in the dataset represent 447 different STs. Moreover, up to 50% of the strains belonged to one of 35 (out of a possible 43) Pneumococcal Molecular Epidemiology Network (PMEN) clones [including single and double locus variants (McGee et al., 2001)]. PMEN clones are resistant to one or more antibiotics in wide clinical use and dominate the population of antibiotic-resistant pneumococci. Globally susceptible clones known to be important in disease are also included in the PMEN clone dataset (https://www.pneumogen.net/pmen/, last accessed September 15, 2020). Further, 2557 strains in the dataset were assigned to one of the 169 Global Pneumococcal Sequence Clusters (GPSCs) and, among these strains, 1096 could be classified as belonging to one of the 35 dominant GPSCs (Gladstone et al., 2019). From a total of 46 serogroups (numbered 1–48) described to date (numbers 26 and 30 are not in use) (Lund and Henrichsen, 1978), 3573 strains belonged to one of 40 serogroups. In addition, the dataset included 4 non-encapsulated laboratory mutants and 426 non-typeable (NT) isolates (**Table S1**).

In agreement with data reported in previous studies (Ramirez et al., 1999; Brueggemann et al., 2017), PPHs were seen to be widely distributed across different *S. pneumoniae* isolates. Indeed, only 434 strains in the dataset (10.8%) (highlighted with a red background in **Table S1**) appeared to lack temperate phages. In addition, among the 126 RefSeq strains with complete (or near complete) genomes (see above), only 26 (20.6%) lacked any discernible prophage. On the basis of their Ints, more than 7000 putative PPHs (including putatively full-length and partial prophage sequences) were found in the dataset. Visual inspection of bacterial genes flanking the prophages revealed up to 26 different integration sites (**Table 1**). PPHs were clustered into 26 groups [named from PPH005 to PPH130 (in steps of 5)] according to their purported insertion sites, and arranged in order using the genome of the non-lysogenic *S. pneumoniae* D39 (Acc. No. NC_008533.2) as a reference. It is worth noting that many more complete Ints (7234) than endolysins (1210) could be identified. In addition, the proportion of partial sequences was much higher for the endolysin genes than the Ints genes. Thus, 42% of the endolysins were found incomplete (887 incomplete out of 2097 total proteins), whereas only 7% of the Ints were apparently incomplete or partly deleted (548 out of a total of 7782) (**Table 1**). This might be because endolysin-coding genes are among those known to be difficult to assemble from short read data due to the presence of choline-binding motif clusters (Croucher et al., 2017).

**Table 1:** Number and distribution of integrases and endolysins in the pneumococcal prophages (PPHs) analyzed.

PPH group	Integrases					Endolysins			
	No.^a^	Alleles (No.)	Size (aa)	Identity (%)	Incomplete	No.	Alleles (No.)	Size (aa)	Incomplete
005	1090 (21)	34	388	>86.5	196				
010	938 (35)	35	382	>95.8	21	286	86	318	102
015	486 (25)	30	380	>96.8	265	60	32	318	457
020	40 (4)	3	388	>58.6^b^	0				
025	1 (1)	1	388	–	0				
030	1151 (25)	17	388	>95.8	52				
035	1 (1)	1	382	–	0	1	1	318	0
040	1 (1)	1	375	–	0	1	1	318	0
045	674 (10)	16	406	>70.0^c^	1				
050	1 (1)	1	375	–	0	1	1	318	0
055	121 (3)	6	388	>98.9	4				
060	5 (2)	2	405	>99.5	0				
065	20 (3)	3	380	>99.4	0	22^d^	1	314	0
070	170 (11)	5	387	>98.7	0				
075	1 (1)	1	375	–	0	1	1	318	0
080	1376 (22)	61	375	>96.7	7	586	165	318	180
						1^e^	1	288	0
085	70 (3)	8	475	>99.1	0	10	9	318	13
090	34 (5)	18	375−481	>17.3	2	6	5	318	4
095	4 (1)	1	388	–	0				
100	885 (5)	56	481	>94.3	1	220	71	318	131
105	6 (1)	4	381	>95.7	0	6^d^	4	334	0
110	47 (1)	3	388	>99.4	0				
115	109 (4)	7	388	>98.1	1				
120	1 (1)	1	479	–	0	1^f^	1	328	0
125	1 (1)	1	475	–	0	7	5	318	0
130	1 (1)	1	382	–	0	1	1	318	0
		(316)					(385)		
Total	7234 (189)	293^g^			548	1210	362^h^		887

a Figures in parentheses correspond to the number of integrases found in 100 complete genomes (176) and 13 contigs of different isolates (13).

b Alleles WP_001866856 and WP_033705527 are 99.7% identical.

c Amino acid identity reached >89.1% when allele WP_050271463 was excluded from alignments.

d Lysozyme (= muramidase; EC 3.2.1.17) of the *Glyco_hydro_25* (PF01183) family instead of NAM-amidase.

e Putative NAM-amidase, but with a cysteine, histidine-dependent amidohydrolase/peptidase (*CHAP*; PF05257) domain.

f Putative NAM-amidase but with a divergent *Amidase_2* domain.

g Due to allele redundancy between different PPH groups, the actual number of Int alleles is 293 rather than 315.

h Corresponds to different prophage-encoded endolysins (357 NAM-amidases plus 5 muramidases).

In sharp contrast to the moderate number of endolysins compared to Ints in the current dataset, the number of different PPH endolysin alleles (362) was similar to that of the Int alleles (293). This strongly suggests that the PPH endolysin-coding gene shows great genetic variability. Nevertheless, 11 PPH groups, namely 005, 020, 025, 030, 045, 055, 060, 070, 095, 110, and 115, appeared to completely lack an endolysin gene and thus were understood to represent incomplete (or defective) prophages. Taking into account the number of full Ints, these incomplete prophages correspond to nearly half (47%) of the total PPHs.

### PPH Integrases

On the basis of amino acid (aa) sequence similarities and catalytic residues, site-specific Ints (recombinases) can be classified into two major families: the tyrosine or serine families (Groth and Calos, 2004). These have different structures and functional mechanisms indicating that they have evolved separately. Thus, serine Ints are usually larger than tyrosine Ints, and the aa residues important for catalysis and structure, as well as their locations, can be very different. For example, the N-terminal domain of tyrosine Ints is involved in binding the arm-type sites of *attP* and the C-terminal domains involved in catalysis, whereas in serine Ints the opposite is true (Groth and Calos, 2004; Van Duyne and Rutherford, 2013).

**Table S2** shows the complete catalog of the Ints found in this study. Excluding the prophages of the PPH090 group, which will be analyzed separately, only three Ints were found in more than one PPH group. These corresponded to the most frequent type of each group, i.e., WP_00876735 (in PPH010 and PPH130), WP_00266847 (in PPH040, PPH075, and PPH080), and WP_000704678 (in PPH055, PPH095, and PPH110). This observation agrees with the common idea that strong (albeit not absolute) specificity exists between prophage Ints and *attB*/*attP* sequences. For example, it has long been recognized that Int-mediated recombination occurs between *attP* of phage λ and the secondary attachment sites in the host genome, although the frequency is low (Shimada et al., 1975). In the present work, sequence identities between Ints were usually >90% within each PPH group. The PPHs contained either tyrosine or serine Ints, although PPHs with tyrosine Ints were 6.5 times more common (6243) than the latter (957) in the dataset (the Ints of PPH090 were not taken into account) (**Table 2**). Serine Ints (groups PPH085, PPH100, PPH120, and PPH125) were much more similar to each other than were the tyrosine Ints (**Table 3**). Among the tyrosine Ints, those from groups PPH010/PPH130 and PPH035 diverged greatly from the rest.

**Table 2:** Active site residues of PPH tyrosine and serine recombinases^a^.

Name (amino acid residues)	Amino acid residue and position															
Tyrosine integrases^b^																
λ Int (356)	R212	D215	K235	H308	R311	H333	**Y342**									
PPH005 (388)	R220	E223	K255	H315	R318	H353	**Y363**									
PPH010/PPH130 (382)	R212	D215	K252	H327	R330	H353	**Y362**									
PPH015 (380)	R218	E221	K255	H324	R327	H350	**Y360**									
PPH020 (388)	R223	E226	K258	H330	R333	H356	**Y366**									
PPH025 (388)	R220	E223	K255	H327	R330	H353	**Y363**									
PPH030 (388)	R223	E226	K258	H330	R333	H356	**Y366**									
PPH035 (382)	R220	D223	K252	H327	R330	H353	**Y362**									
PPH040/PPH075/PPH080 (375)	R211	E214	K247	H320	R323	H346	**Y356**									
PPH045 (406)	R234	E237	K269	H349	R352	H375	**Y384**									
PPH050 (375)	R211	E214	K247	H320	R323	H346	**Y356**									
PPH055/PPH095/PPH110 (388)	R220	E223	K255	H327	R330	H353	**Y363**									
PPH060 (405)	R234	E237	K269	H349	R352	H375	**Y384**									
PPH065 (380)	R217	E219	K249	H326	R329	H352	**Y362**									
PPH070 (387)	R222	E235	K257	H329	R332	H355	**Y365**									
PPH105 (381)	R208	E211	K246	H323	R326	H349	**Y359**									
PPH115 (388)	R220	E223	K255	H327	R330	H353	**Y363**									
Serine integrases^c^																
TP901-1 Int (485)	Y8	R10	**S12**	Q26	V41	D47	R57	P58	D73	V77	D81	R82	L83	R85	G119	E133
PPH085 (475)	Y10	R12	**S14**	Q28	I43	D49	R59	P60	D75	V79	D83	R84	L85	R87	G121	E135
PPH100 (481)	Y10	R12	**S14**	Q28	V43	D49	R59	P60	D75	V79	D83	R84	L85	R87	G121	E135
PPH120 (479)	Y7	R9	**S11**	Q25	I40	D46	R56	P57	N72	V76	K80	R81	L82	R84	G118	D132
PPH125 (475)	Y10	R12	**S14**	Q28	I43	D49	R59	P60	D75	V79	D83	R84	L85	R87	G121	E135

a PPH090 integrases were not included.

b The active site residues of the phage λ integrase (λ Int; Acc. No. P03700) have been previously described (Gibb et al., 2010). The catalytic nucleophile, Tyr342, is boldface. The accession numbers for the other tyrosine integrases studied here are: PPH005, WP_000704676; PPH010/PPH130, WP_000876735; PPH015, WP_000266841; PPH020, WP_033705527; PPH025, WP_000704686; PPH030, WP_000704664; PPH035, WP_000876736; PPH040/PPH075/PPH080, WP_000266847; PPH045, WP_000219075; PPH050, WP_061816163; PPH055/PPH095/PPH110, WP_000704678; PPH060, WP_001863308; PPH065, WP_000266851; PPH070, WP_001021836; PPH105, WP_054368747; and PPH115, WP_001866671.

c The active site residues of the *Lactococcus lactis* phage TP901-1 integrase (TP901-1 Int; Acc. No. CAA59475) have been previously described (Yuan et al., 2008). The catalytic nucleophile, Ser12, is boldface. The accession numbers for the other serine integrases studied are: PPH085, WP_050199652; PPH100, WP_024478469; PPH120, WP_130892475; and PPH125, WP_023396450.

**Table 3:** Sequence similarities among tyrosine and serine integrases of PPHs ^a^.

	Integrases	2	3	4	5	6	7	8	9	10	11	12	13	14		15	16
PPH group	Tyrosine integrases																
005	(1) WP_000704676	−7	−26	−145	≤−180	−143	−7	−21	−11	−21	≤−180	−7	−14		−122	−55	≤−180
010/130	(2) WP_000876735		−6	−5	−4	−3	≤−180	−3	−6	−3	−4	−10	−4		−2	−12	−5
015	(3) WP_000266841			−30	−25	−27	−5	−112	−9	−110	−25	−8	−65		−17	−26	−25
020	(4) WP_033705527				−146	≤−180	−4	−21	−16	−20	−146	−13	−18		−154	−56	−148
025	(5) WP_000704686					−144	−4	−21	−11	−21	≤−180	−6	−14		−123	−57	≤−180
030	(6) WP_000704664						−3	−25	−13	−24	−144	−12	−18		−151	−54	−146
035	(7) WP_000876736							−2	−6	−2	−4	−10	−4		NS	−12	−5
040/075/080	(8) WP_000266847								−12	≤−180	−21	−12	−63		−60	−31	−22
045	(9) WP_000219075									−11	−11	≤−180	−9		−20	−24	−10
050	(10) WP_061816163										−21	−12	−61		−19	−31	−22
055/095/110	(11) WP_000704678											−6	−14		−123	−57	≤−180
060	(12) WP_001863308												−9		−16	−24	−6
065	(13) WP_000266851														−18	−17	−21
070	(14) WP_001021836															−50	−120
105	(15) WP_054368747																−56
115	(16) WP_001866671																
	Serine integrases																
085	(1) WP_050199652	≤−170	−146	≤−180													
100	(2) WP_024478469		−179	−171													
120	(3) WP_130892475			−148													
125	(4) WP_023396450																

a Figures correspond to Log_10_ *E* values calculated by pairwise alignments. NS, not significant.

### PPH Endolysins

Endolysins encoded by virulent (lytic) phages infecting *S. pneumoniae* have been extensively reviewed (López and García, 2004; García et al., 2005; Galán-Bartual et al., 2015; Maestro and Sanz, 2016; Vázquez et al., 2018). Briefly, three biochemically and structurally different peptidoglycan hydrolases of phage origin have been described so far. Two of them are choline-binding proteins: the NAM-amidase Pal (YP_004306947; 296 aa), with an *Amidase_5* domain (PF05382), and the Cpl-1 lysozyme (=muramidase) (NP_044837; 339 aa) with a *Glyco_hydro_25* (PF01183) domain. Both enzymes contain a C-terminal cell wall-binding domain composed of six choline-binding repeats (*Choline_bind_1*; PF01473). Cpl-7 (YP_009623604; 342 aa) is a choline-independent muramidase that differs from Cpl-1 in the C-terminal domain responsible for peptidoglycan binding (*CW_7* repeats; PF08230). Pal is encoded by phage Dp-1, whereas Cpl-1 and Cpl-7 are the endolysins of phages Cp-1 and Cp-7, respectively. The endolysin from a recently isolated virulent podovirus very similar to Cp-1 (SOCP) (Ouennane et al., 2015) (NP_044837; 339 aa), is a muramidase 100% identical to Cpl-1. Similarly, the NAM-amidase (AQY55407; 295 aa) of the MS1 pneumococcal siphovirus (Kot et al., 2017; Silva et al., 2020) is 81% identical (89% similar) to Pal.

Taking into account that only a few virulent *S. pneumoniae-*infecting phages have been currently isolated, the diversity of their endolysins contrasts with the lytic enzymes encoded by PPHs. The latter are 318 aa-long, belong to the *Amidase_2* family (PF01510) of NAM-amidases, and contain six *Choline_bind_1* repeats in the C-terminal domain (Morales et al., 2010; Morales et al., 2015). Remarkably, in addition to having the same length (957 bp), the known phage genes coding for these endolysins (*lytA*_PPH_) are closely related (85–92% identity) to *lytA_Spn_*, which encodes the major pneumococcal autolysin —also a NAM-amidase of the *Amidase_2* family (Morales et al., 2010). LytA*_Spn_* is a well-known virulence factor that plays a role(s) during different steps of infection (Canvin et al., 1995; Ramos-Sevillano et al., 2015; Ramos-Sevillano et al., 2016; Corsini et al., 2021). Taking into account the strong similarities between bacterial and phage NAM-amidases, the latter may also be important in pneumococcal pathogenesis.

The current notion of an exclusive presence for *lytA*-like genes among PPHs could not be fully confirmed in the present study. As already mentioned, 1210 full-length endolysins were identified in the dataset (**Table 1** and **Table S3**). Among those, 29 proteins (≈2.5%) did not correspond to NAM-amidases of the *Amidase_2* family. There were 28 proteins homologous to the Cpl-1 lysozyme, with one containing a cysteine, histidine-dependent amidohydrolase/peptidase (CHAP) domain (PF05257) instead of an *Amidase_2* domain at the N-terminal moiety. The lysozymes were endolysins of PPH065 (22 identical proteins; WP_000739159) and PPH105 (6 proteins; 4 alleles: WP_054365577, WP_054368721, WP_054380492, and WP_054392100), which are 314 aa- and 334 aa-long respectively. Notably, all strains harboring PPH065 are members of GPSC19 and have serotype 22F (the only exception is strain 2245STDY6178828 which belongs to serotype 42) (see below).

The main differences between Cpl-1 and the PPH lysozymes detected in the present study are located in the linker region connecting the N- and the C-terminal domains. Thus, in Cpl-1, the linker (189-DDEEDDKPKTA-199) (Hermoso et al., 2003) is longer than (and different to) that of the newly discovered endolysins (188-DDEEAKAK-195). Moreover, WP_000739159 lacks the fourth *Choline_bind_1* repeat that forms part of the C-terminal domain of the enzyme, and which is responsible for binding the enzyme to the cell wall. A detailed analysis of other PPHs (156 additional genomes, either complete or not) revealed four additional examples of 334 aa-long Cpl-1 homologs, specifically those encoded by prophages IPP16, IPP25, IPP27, and by a nameless prophage harbored by *S. pneumoniae* strain R34-3131 (**Table S4**). The latter prophage (which belongs to the PPH105 family) was already present in the dataset (**Table S1**).

The CHAP domain-containing endolysin (WP_057595562; 288 aa) found in the genome of *S. pneumoniae* strain SMRU392 (serotype 35F) is encoded by one of the PPH080 group of phages, and is identical to that of prophage 33888. The sequence of the latter was recently reported in an independent study (van Tonder et al., 2019) (**Table S4**). Since the host strain of prophage 33888 was the same as in the present study (SMRU392), and the Ints were also identical (WP_ 050256063), both prophages are probably the same. The WP_057595562 PPH endolysin is 63% identical (77% similar) to Skl, a proven NAM-amidase (WP_033686260; 288 aa) of an unnamed temperate phage of *Streptococcus mitis* SK137 (Llull et al., 2006). The complete genomic sequence of, presumably, the same *S. mitis* prophage (now designated as Javan331; MK448732) has recently been reported (Rezaei Javan et al., 2019). Remarkably, the existence of putative CHAP-endolysins encoded by those prophages was not mentioned in any study from other laboratories.

Among the 1181 endolysins with a predicted *Amidase_2* domain, the presence of one atypical protein was noted. This protein (WP_130892444; PPH120) is a 328 aa-long, putative NAM-amidase with a divergent *Amidase_2* domain. It is an endolysin encoded by the 36.7 kb-long prophage inserted into PPH010_4, giving rise to PPH120 (see below). Searches for proteins very similar (≥87% identity) to WP_13089244 revealed several endolysins of the same length encoded by prophages of three members of the Mitis group streptococci, i.e., *Streptococcus pseudopneumoniae, S. mitis*, and *Streptococcus oralis*. Specifically, these prophages are 277_SPSE, 289_SPSE, and 380_SPSE (WP_049511163) from *S. pseudopneumoniae* (Roach et al., 2015), *S. mitis* strains SK564 (WP_000238871) (Kilian et al., 2014) and DD22 (WP_061864892) (Denapaite et al., 2016), *S. oralis* U-o11 (Javan367; QBX17588) (Rezaei Javan et al., 2019), and *S. oralis* subsp. *tigurinus* 859 (WP_084868230) (Diene et al., 2016). Sequence alignments also revealed the variant domain of WP_130892444 to be remarkably similar to that of the *Amidase_2* domain of LysGH15, the endolysin encoded by a myovirus phage (GH15) that infects *Staphylococcus aureus* (Gu et al., 2011). This lysin possesses a modular structure containing an N-terminal CHAP domain, a central *Amidase_2* domain, and a C-terminal SH3_5 (PF08460) bacterial-binding domain. Elucidation of the crystal structure of the *Amidase_2* domain of LysGH15 (Gu et al., 2014) revealed the aa residues involved in Zn^2+^ binding (H214, H324, and C332), catalysis (E282, and T330), as well as other important residues (W263, and N75), to be conserved in WP_130892444 at comparable positions (**Figure S1**). In sharp contrast, some of the equivalent residues in the NAM-amidase of *S. pneumoniae* TIGR4 (Mellroth et al., 2014; Li et al., 2015)—namely H26 and D149, as zinc ligands, and the catalytic residue H147—differed from those of WP_130892444 (and LysGH15) but were fully conserved among the widespread 318 aa-long NAM-amidases (**Figure S1**).

### Insights Into the PPH Genomes and Identification of Their Attachment Sites

Only four *att* core sequences for PPHs were reported in studies published up until 2009 (Gindreau et al., 2000; Obregón et al., 2003b; Romero et al., 2009a), and additional core sequences have only been seldom reported in more recent papers (Càmara et al., 2018; Garriss and Henriques-Normark, 2020). To the best of our knowledge, only seven different *attB* core sequences have been previously described (**Table S5**). Remarkably, genome examination of many PPHs currently deposited in public databases (131 out of 158; last accessed, January 30, 2021) allow for no precise identification of the *attP* core site because most of the reported phage genomes only include the DNA region running from the first nucleotide of the *int* gene to the last one of the endolysin-coding gene (even though the definition was that of ‘complete genome’). Further, they lack the corresponding intermediate sequence where *attP* ought to be located (**Table S4**).

To gain insight into the sequences and locations of the integration sites of the PPHs described in the present study, a detailed analysis of all the PPHs harbored by 126 strains with near complete (chromosome assembly level) genomes was performed. The strains included in this subset represent 88 different STs, 27 PMEN clones, 66 GPSCs, and 27 different serotypes. In addition, the dataset included one non-encapsulated laboratory mutant and four NT isolates (**Table S6**). As mentioned above, 100 out of 126 strains (79.4%) were lysogenic and fulfilled the above assembly requirements in the original dataset. Fifty one isolates harbored only one PPH per genome whereas 49 were polylysogenic, and one of them (strain GPSC72) contained up to five different prophages (**Table S6**). Taking advantage of previously determined locations of *int* genes, the genomes of these strains were examined in detail and the sequences corresponding to the *attL* and *attR* sites of 176 prophages belonging to 20 different PPH groups recorded. The sequences and locations for the remaining six groups (PPH035, PPH065, PPH090, PPH095, PPH105, and PPH110) were estimated using the partial genomes (either at the contig or scaffold assembly level) of nine additional strains included in the dataset. Prophage genomes were defined as the DNA sequence running from the first nucleotide of *attL* to the last one preceding *attR.* The *attL* sequences (obviously identical or near identical to their corresponding *attR* within each PPH group) were used as queries to map the corresponding *attB* sites on the *S. pneumoniae* D39 genome (**Table 4**). With the exception of PPH130, the core sequence of 24 different *attB* could be determined. As mentioned above, seven of them had been already reported, namely, PPH005, PPH010, PPH015, PPH035, PPH080, PPH085, and PPH100 (compare the data of **Table 4** with those of **Table S5**). With the exception of PPH075, PPH125 and PPH130, in which *attB* sites appear to be located inside insertion sequences (ISs), the precise chromosomal location of the different *attB* sites could be established. Although many PPH insertion sites are located in intergenic spaces (something typical among prophages), six core attachment sites mapped within the 3’ region of genes annotated for the D39 genome, and included the termination codon (TAA) of the gene where the prophage was integrated (**Table 4**). In addition, *attB*_PPH035_ was found to partially overlap (but apparently not interrupt) the 3’ part of *ccnB* encoding csRNA2, one of the five small non-coding csRNAs (cia-dependent small RNAs) that form part of the two-component regulatory system CiaRH (Sinha et al., 2019). It should be underlined that *ccnB* is not annotated for the *S. pneumoniae* D39 genome. On the contrary, seven prophage groups potentially interrupt gene translation: 1) the core integration site of PPH010 viruses (*attB*_PPH010_), previously reported to lie between SPD_RS00115 and SPD_RS00120 (Romero et al., 2009a) but actually located in *ccnC* coding for csRNA3 (Furi et al., 2019); 2) PPH015, which integrates into SPD_RS00125 encoding the signal recognition particle sRNA involved in membrane protein targeting (Steinberg et al., 2018); 3) PPH040, which integrates into SPD_RS01460, potentially encoding an intramembrane metalloprotease of the CAAX proteases and bacteriocin-processing enzymes (CPBP) family (Pei et al., 2011); 4) PPH050, inserted into SPD_RS01935 encoding a frameshifted choline-binding protein (CbpG) in D39 (Frolet et al., 2010); 5) a gene (SPD_RS09795) encoding UlaR [a transcriptional activator of the *ula* operon in the presence of ascorbic acid (Afzal et al., 2015)], which may be inactivated by the insertion of bacteriophages of the PPH095 group; 6) PPH100, which integrates into SPD_RS09885 encoding ComGC, the major subunit of the competence pilus (Laurenceau et al., 2015); 7) and bacteriophages of the PPH120 group, which disrupt SPD_RS10405, i.e., the gene encoding for the PcpA choline-binding protein (Sánchez-Beato et al., 1998) (now an important component of several new protein-based, pneumococcal vaccines currently under evaluation) (Masomian et al., 2020).

**Table 4:** Localization of *attB* sites for *S. pneumoniae* temperate prophage integration.

Prophage	*attB* (5’→3’)^a^	Flanking locus tags (SPD_RS) (Product)
PPH005	2935 TTAGCACTTTATCCCTTTTTGTGTTA 2960	00015 (DUF951 family protein)/00020 (redox-regulated ATPase YchF)
PPH010	24016 CTTTTTCATAATAATCTCCCT 24036^b^	00115 (adenylosuccinate synthase/00120 (nucleoside deaminase)
PPH015	24694 TTGTGTGCTCTTTTTTTCGTGC 24715	00125 (signal recognition particle sRNA small type)
PPH020	86374 TACAACAAAATGTTG **TAA** TATTT 86396	00445 (30S ribosomal protein S4)
PPH025	163831 ATTCCTTTACAA 163842	00880 (response regulator transcription factor)/00885 (hypothetical protein)
PPH030	179128 ATTATACTACAAAATCGGCCTTTT 179151	00970 (magnesium transporter CorA family protein)/00975 (excinuclease ABC subunit UvrA)
PPH035	231378 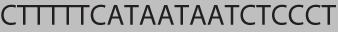 TAACTCCACCCAATCAGGTGGAGTTTTTTAGCTCTATTTCAGGCTTTTGGGGACTATTCTAAAAATAATTTTTCGATATTTTTCGGTATTTTTCGGATTTTGGTCGGGGAATTGGCGGGGACTTTTT 231525^c^	01305 (GNAT family N-acetyltransferase)/01310 (type I toxin-antitoxin system Fst family toxin)
PPH040	267293 GGTCTTTTTACTTGCCG 267309	01460 (CPBP family intramembrane metalloprotease)
PPH045	273279 GTAAAGCATCACAATTTAGTAAACGT **TAA** T 273308	01495 (30S ribosomal protein S9)
PPH050	358211 AGTCAAGAACTATTT 358225	01935 (choline-binding protein CbpG; frameshifted)
PPH055	619559 CATATTATTTTGAAAT 619574	03210 (DUF3165 family protein)/03215 (lactococcin 972 family bacteriocin)
PPH060	693388 AACCAGATCTTAAGAAAGCTCGTAAAG 693414^d^	03620 (hypothetical protein)/03630 (hypothetical protein)
PPH065	1006452 CTCTTAAAGACGCTGTTAAA **TAA** T 1006475	05350 (HU family DNA-binding protein)
PPH070	1027141 TACAACCTTAAAAAA **TAA** 1027158	05425 (phosphopyruvate hydratase)
PPH075	ATGCCGATGAATTATAA	Prophage located 3’ of SPD_RS06250 (cyclically-permuted mutarotase family protein)
PPH080	1415148 **TTA** TAATTCATCCGC 1415162	07415 (DNA-binding protein WhiA)
PPH085	1712415 TTCCTCCTACTTATCTATTCGTAG 1712438	09110 (single-stranded DNA-binding protein)/09115 (SDR family NAD(P)-dependent oxidoreductase)
PPH090	1729602 **TTA** TTTTACTGTAATCAAGCCATCTGGCTCTACTGTGAATTCTGGC 1729647	09250 (*N*-acetylmuramoyl-L-alanine amidase family protein)
PPH095	1826920 CATTACGAAATATATT 1826935	09795 (transcription anti-terminator)
PPH100	1841058 ACCAACATCTCCACCAA 1841074	09885 (*comG* operon protein ComGC)
PPH105	1849319 TATGGTATAA 1849328^d^	09920 (tRNA guanosine(34) transglycosylase Tgt)/09925 (DUF975 family protein)
PPH110	1901612 ATAA 1901615^e^	10215 (methyltransferase domain-containing protein)/10225 (membrane protein)
PPH115	1911323 ACTTGAAATAAAGCGCATTTCTCTATA 1911349	10255 (sugar ABC transporter permease)/10265 (DUF1189 domain-containing protein)
PPH120	1933870 ATTCGTTTAAGTAATACCATAAACCTTTGTCTTTAACCCAACCAGTAGCCA 1933920^f^	10405 (Choline-binding protein PcpA)
PPH125	ATGTTATTTCTCTCGTTACAAATTACAACCTTAAAAAA **TAA**	Prophage located 3’ of SPD_RS10665 (tRNA dihydrouridine synthase DusB). The predicted *attB* forms part of IS*Spn5* (IS1380 family).
PPH130	Not known	Prophage located 5’ of SPD_RS10885 encoding a transposase.

a The coordinates correspond to those in the *S. pneumoniae* D39 genome (NC_008533.2). This strain was recently renamed ‘D39W’ (Sinha et al., 2019) and showed some differences to another cultivar of the same strain (D39V) (Slager et al., 2018). Termination codons (TAA) are bold and underlined.

b This *attB* is located inside the *ccnC* gene (23,967−24,065) encoding a small non-coding csRNA (cia-dependent small RNA) named csRNA3 (Sinha et al., 2019). This gene is not annotated for the pneumococcal D39 genome. Two additional potential *attB* sequences are present in the *S. pneumoniae* D39 genome, i.e., between SPD_RS01305 and SPD_RS01310 (231,378–231,398) (see shadowed sequence in PPH035), and between SPD_RS01320 and SPD_RS01325 (233,750–233,770).

c The shadowed sequence is identical to that of *attB_PPH010_*. The part of the sequence overlapping the 3’ end of the ccnB gene (231,331–231,427) coding for csRNA1 (Sinha et al., 2019), is underlined. This gene is not annotated for the pneumococcal D39 genome.

d Another potential *attB* sequence (273,258-AACCAGgTCTTAAGAAAGCTCGTAAAG-273,284) is present in the *S. pneumoniae* D39 genome, i.e., inside the SPD_RS01495 gene, encoding the 30S ribosomal protein S9.

e Identical sequences are present at many other positions.

f Seven additional potential *attB* sequences are present in this gene.

**Table S6** attempts to organize the different PPHs in a manner that takes into account their wide genetic diversity. For this, a concept of ‘equivalent PPH genomes’ was followed, i.e., genomes that overlapped for ≥90% of their total length and showed ≥90% sequence identity were considered ‘equivalent’, and only one of them was used in further comparisons. **Figure S2** shows a comparison of the different PPH genomes. PPH095 and PPH110 are not depicted since, although they inserted into two different *attB*s, these PPHs were equivalent to PPH055 (see below). Due to the evident sequence variability of prophage genomes, pairwise nucleotide alignments were initially performed only among PPHs of the same group. Several main conclusions were then drawn: 1) *int* genes were present in every prophage found, but this was not the case for endolysin-coding genes; 2) two major PPH groups were recognized, one with genome lengths ranging from ≈10–20 kb on one side (e.g., PPH005 and PPH030 groups), and the other from ≈35–45 kb on the other side (e.g., PPH010 and PPH080 groups); 3) major differences can still be seen among the members of a PPH group (e.g., PPH015, PPH020, PPH045 and PPH090 show large differences); 5) several PPHs with genomes of 51–92 kb appear to result from recombination events between two different prophages, e.g., *i*) PPH075 is the result of the insertion of an unknown defective prophage (12,227 bp) into a PPH085_5 equivalent. This defective prophage is flanked by a 33 bp-long sequence (5’-CCCTAGACTTGAAATAAAGCGCATTTCTCTATA-3’) located at positions 32,873–32,905 and 45,100–45,132 of the PPH075 genome; a near-identical sequence (1,911,317-TCCTAGACTTGAAATAAAGCGCATTTCTCTATA-1,911,349) may contain part of the promoter region of SPD_RS10265 (*malA*) in the D39 genome (Nieto et al., 1997); *ii*) PPH080_11 appears to result from the integration of a PPH010-like prophage into a PPH080_3 equivalent; as expected, the insertion of the PPH010-like phage occurs in the sequence 5’-CTTTTTCATAATAATCTCCCT-3’ at positions 2069–2089 and 32,251–32,271 of the PPH080_11 genome; *iii*) the insertion and rearrangement of a PPH010_4-equivalent into a previously unknown prophage gives rise to PPH120, possibly with the assistance of two ISs (IS*Spn5* and IS*1167*) and the addition of a gene cluster encoding several tRNAs. Sequence comparison revealed this prophage to be very similar (82–85% query coverage and >95% nucleotide sequence identity) to two *Streptococcus anginosus* prophages, namely Javan83 (Rezaei Javan et al., 2019) and SA01 (van der Kamp et al., 2020); interestingly, the latter two prophages appear to harbor no tRNA genes. In addition, both encode an endolysin 76% identical (88% similar) to Cpl-7 (see above), whereas, as already discussed, the new component of PPH120 codes for a peculiar 328 aa-long NAM-amidase of the *Amidase_2* family of proteins; *iv*) a tandem insertion of two near identical PPHs (PPH080_1A and PPH080_1B) gives rise to the 92 kb-long hybrid prophage PPH080_1AB.

Among the various prophage identification tools, we used the PHASTER program to analyze the 109 genomes included in **Table S6** (186 PPH). Up to 263 putative PPHs were predicted; 40 of them were designated as ‘intact’ by the program and actually corresponded to real prophages (**Table S6**). The predicted limits of the PPHs did match the real coordinates in only few cases (e.g., PPH030 from strain SP64 was almost correctly located (real: 177,043–189,925; predicted: 177,043–189,926). Unfortunately, in most instances, this was not the case. For example, in strain GPSC47 (NZ_LR216060), PPH010_9 is located between coordinates 3152 and 36,649 whereas PHASTER predicted the range 1771–42,683). Of note, the existence of PPHs of the groups 010, 015, 080, and 110 was well predicted, whereas that of those belonging to groups 020, 045 and 115 were not (**Table S7**). In our hands, the sensitivity of PHASTER (0.74) was similar to that previously reported (Lopes de Sousa et al., 2018; Reis-Cunha et al., 2019).

**Figure 1:**
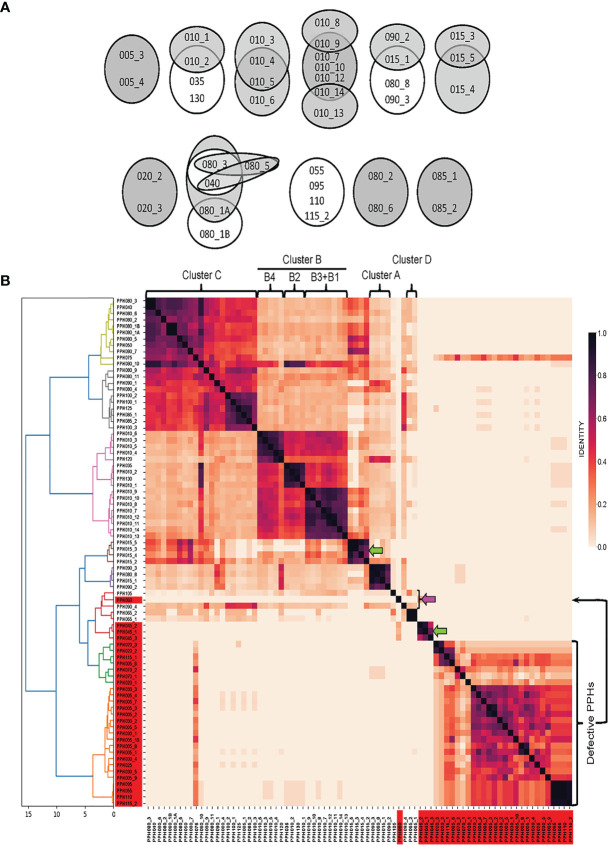
Sequence similarities between PPHs genomes. **(A)** Venn diagrams showing equivalent or very similar pneumococcal prophages. Two PPH genomes were considered ‘equivalent’ when they overlapped for ≥90% of their length and showed ≥90% sequence identity (shown on a white background). When two genomes overlapped between 80 and 89% of their length, and nucleotide identities were ≥90%, the corresponding PPHs were considered as ‘very similar’. They are shown on a gray background. **(B)**. wGRR similarity heatmap depicting the amino acid identity shared between pairs of prophage sequences (either full-length or defective prophages). Different colors were applied to clusters showing distance thresholds > 4. For agglomerative hierarchical clustering, distances are shown in the X axis. The *dendrogram* and *heatmap* python tools of the scipy.cluster.hierarchy library were used to depict final agglomerative hierarchical clustering and heatmap plots, respectively. Green and pink arrows point to new clusters or singletons respectively. The correspondence between PPHs and the cluster organization of pneumococcal prophages previously reported (Brueggemann et al., 2017; Rezaei Javan et al., 2019) is indicated at the upper part of this panel. See **Table S4** for a proposed correlation between previously described pneumococcal prophages and PPH groups.

Pairwise nucleotide alignments were then performed among genomes belonging to different PPH groups. In addition to equivalent PPHs (see above), a second level of similarity was allowed, i.e., those prophages with an overlap of between 80 and 89% of their genomes and showing nucleotide identities of ≥90%. **Figure 1A** shows ‘equivalent’ and ‘very similar’ genomes on white or gray backgrounds respectively. It should be underlined that equivalent prophages were distributed between different PPH groups. Thus, in addition to the equivalency between the defective prophages PPH055, PPH095, PPH110 and PPH115_2 partly mentioned above, other equivalent, putatively complete prophages of different groups were seen: 1) PPH010_2, PPH035 and PPH130; 2) PPH015_1, PPH080_8 and PPH090_3; and 3) PPH040 and PPH080_3. As expected, most of the ‘very similar’ category of prophages corresponded to different members of the same group, as exemplified by members of the PPH010 group such as PPH010_7–PPH010_10 and PPH010_12–PPH010_14 (**Figure 1A**). Pairwise comparison of 80 PPH sequences and a dendrogram depicted seven major prophage clusters, and three singletons (**Figure 1B**). Four of the clusters matched those described by Brueggemann et al. (2017) and one more to the defective prophages mentioned by Rezaei Javan et al. (2019) that were recognized as separate entities from full-length PPHs. The remaining PPHs (either clustered or not) correspond to previously undescribed prophages.

### Other Features of Pneumococcal Prophages

Many prophages from both Gram-negative and Gram-positive bacteria integrate into tRNA genes (Williams, 2002) although this is not the case for PPHs (see above). Nevertheless, sequence analysis revealed the presence of potential tRNA genes in some PPH genomes (**Table S8**). Of note, PPH120 has nine tRNA genes located at the right end of the prophage, whereas other PPHs have these genes located closer to the middle. A search of PPH genomes reported elsewhere revealed that five previously sequenced phages (namely, IP8, IP16, IP25, IP26, and IP27) also harbor a tRNA-Ser gene, a feature not mentioned in previous reports (**Table S8**).

PblB may be an important virulence determinant in some PPHs. PblB has been described as a phage minor tail protein (a putative anti-receptor) that behaves as an adhesin mediating the galactose-specific adhesion activity of pneumococci to platelets and human lung epithelial cells *in vitro*. It is also required for nasopharyngeal and lung colonization in a mouse model of infection (Hsieh et al., 2015). The sequence diversity of PblB is notable (Brueggemann et al., 2017). Amino acid sequence alignments in the PPH dataset fully confirmed this (as shown diagramatically in **Figure S3**), even among PPHs of the same group (e.g., in PPH010_7, in which the length of PblB was 2134 aa in strain GPSC97 but 3256 aa in GPSC25). It is also reported that the PblB encoded by the temperate phage SM1 infecting *S. mitis* (1062 aa) (Bensing et al., 2001) is very different to the protein (P15; 1987 aa) previously analyzed in the *S. pneumoniae* NTUH-P15 strain (Hsieh et al., 2015). Moreover, these two proteins differ notably from most of the PblB proteins found in the present study, particularly in their middle regions where different combinations of five different (albeit related) sequence repeats were found (**Figure S3**). PblB-like proteins were encoded by some of the phages belonging to the PPH010, PPH035, PPH040, PPH080, PPH085, and PPH100 groups. Willner et al. showed that the *S. mitis* prophage SM1 can be induced by commonly ingested substances and that the oral cavity is indeed a reservoir of *pblA* and *pblB* genes (Willner et al., 2011).

First described in the Gram-negative bacterium *Dichelobacter nodosus* (an ovine footrot pathogen), virus-associated protein E (VapE*_Dno_*) has been proposed involved in virulence (Billington et al., 1996). Proteins similar to VapE have also been reported encoded by prophages of other microorganisms such as *S. aureus* (Lindsay et al., 1998), *Pseudomonas putida* (Canchaya et al., 2003), *Vibrio parahaemolyticus* (Seguritan et al., 2003), *Staphylococcus epidermidis* (Chen et al., 2013) and *Elizabethkingia anophelis* (Peng et al., 2020), although direct evidence of the involvement of VapE in virulence was provided in neither of these studies. In *S. pneumoniae*, Romero et al. (2009a) first noted that several prophages encoded proteins similar (about 30% identity; *E* value = 10^–34^) to VapE*_Dno_* (also designated as *virE*). More recently, several gene products from defective prophages have been shown similar to a *Streptococcus suis* protein named VapE*_Ssu_* (Rezaei Javan et al., 2019), a protein apparently unrelated in primary structure to VapE*_Dno_* but which has been shown involved in virulence in a mouse model (Ji et al., 2016). The translated nucleotide dataset of PPHs was compared to both VapE*_Dno_* and VapE*_Ssu_* and using an *E* value cutoff of 10^–20^, two sets of putative virulence-associated proteins were found (**Table S9**).

It is well known that bacteria have developed defense mechanisms to help them survive phage attacks. These mechanisms may interfere with the adsorption of the phage onto the cell surface, with phage genome injection, or target the foreign nucleic acid after its injection, etc. (for recent reviews see Labrie et al., 2010; Koonin et al., 2017; Rostøl and Marraffini, 2019). Phages, in turn, have evolved mechanisms to overcome restriction-modification (R-M) systems. One of these is based on the presence in phage genomes (both virulent and temperate) of orphan methyltransferase (Mtase) genes (i.e., without concomitant endonuclease-coding genes) (Murphy et al., 2013). There are three functional classes of type II DNA Mtases that function by transferring a methyl group from *S*-adenosyl-L-methionine to a target nucleotide base, forming either N-6-methyladenine (class I), N-4-methylcytosine (class II), or C-5-methylcytosine (class III). Our laboratory previously reported the existence of putative Mtases-encoding genes in two *S. pneumoniae* temperate phages, namely MM1 (Obregón et al., 2003a) and VO1 (Obregón et al., 2003b). Taking the Mtases of these two phages as query sequences, the complete set of PPH genomes was screened. Interestingly, more than 30 putative orphan Mtases of either the MM1 or VO1 class were detected. None of them was encoded by defective PPHs (**Table S10**).

### PPH090, a Group of Prophages That Integrate Into the *lytA_Spn_* Gene *via* an Int-Independent Mechanism

The proposed core sequence of *attB*_PPH090_ (46 bp-long) mapped to the 3’ end of the *lytA_Spn_* gene overlapping its termination codon (**Table 4**). Consequently, phage integration should not disturb *lytA* translation. In sharp contrast with other PPHs, the most striking feature of prophages of the PPH090 group is that there is no preference for a particular Int (or group of Ints) (**Table S2**). Specifically, different PPHs of the PPH090 group encode Ints of the tyrosine (12 entries) or serine (6 entries) families, some of which are identical to those of other prophage groups: 1) tyrosine Ints [PPH010 (WP_000876726, WP_000876733, WP_000876735, and WP_050199954), PPH015 (WP_023396066, WP_044813689, WP_050127433, W_050203801, and WP_050221901), PPH080 (WP_000266839, WP_000266847, and WP_000266854)]; and 2) serine Ints [PPH100 (WP_024478469, WP_044812715, WP_050203932, WP_050226267, WP_050230313, and WP_050974546)]. These data strongly suggest that PPH integration into *lytA_Spn_* does not require a specific interaction between *attB, attP*, and the Int, and that some other factors may be involved—possibly recombination events between *lytA_Spn_* and *lytA*_PPH_ (see below).

The four PPH090 genomes shown in **Figure S2** encode, respectively, one of three different Ints of the tyrosine family (PPH090_1–PPH090_3) and one Int of the serine family (PPH090_4). Among them, the genome of the NT strain SMRU257 (master record NZ_CKNY01000000) appears to be unique since, in addition to the PPH090_2 genome (39,182 bp; contig NZ_CKNY01000002), it contains a 39,182 bp-long sequence (contig NZ_CKNY01000010) inserted into the *ffs* gene (corresponding to SPD_RS00125 in *S. pneumoniae* D39) (**Table 4**). Moreover, this prophage harbors all the characteristics of a full member of the PPH015 group, including the near identical flanking *attL* and *attR* core sequences (5’-TTGTGTGCTCTTTTTTCGTGC-3’). Remarkably, PPH090_2 and the ‘new’ PPH015 only differ at two nucleotide positions, i.e., 918 (G) and 951 (T) of the 957 bp-long *lytA*_PPH_ and *lytA*_PPH_* genes (the asterisk indicates that the phage and bacterial *lytA* genes were probably chimeric [a hybrid] because they contain sequences of both phage and bacterial origin) (**Figure S4**).

**Figure 2:**
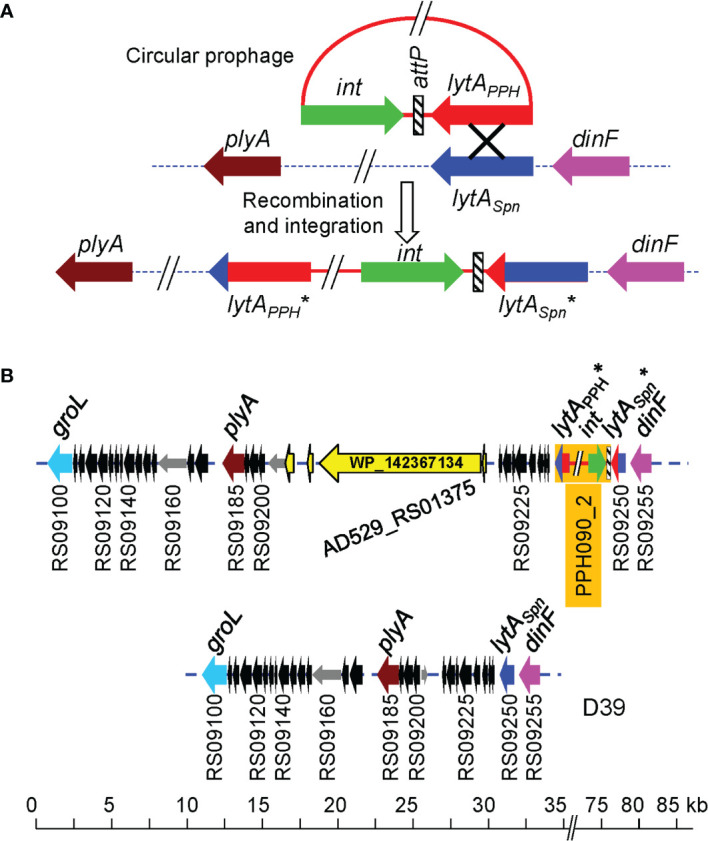
A proposal for phage integration into the *S. pneumoniae* genome after the recombination of *lytA_Spn_* and *lytA*_PPH_. **(A)** The circular phage genome (red) and the pneumococcal chromosome (dotted line) are shown. Genes are drawn as arrows indicating the direction of transcription. An ‘X’ indicates the recombination event. The pneumococcal genes *plyA* and *dinF* that flank the *lytA_Spn_* gene are shown for reference (Morales et al., 2015). The asterisk (*) indicates that the phage and bacterial lytA genes were probably chimeric because they contain sequences of both phage and bacterial origin. **(B)** Novel genomic arrangement of this *plyA—lytA* island in the pneumococcal strain SMRU257. The location of a gene (AD529_RS01375) encoding a large protein (3628 aa residues; WP_142367134) is shown. For comparison, the corresponding DNA region of the control D39 strain is shown at the bottom.

The hypothesis that *lytA*_PPH_ recombines with the *lytA_Spn_* gene and integrates into the pneumococcal genome was tested by making a detailed analysis of both the *lytA* genes and the DNA regions located between *int* and *lytA_Spn_* *, as well as downstream of *lytA*_PPH_*, in the integrated state (**Figure 2A**). **Figure 2B** shows the appropriateness of the hypothesis since, immediately after the termination codon of *lytA*_PPH_*, the sequence was syntenic, *i.e.* shares the same genetic order, with the DNA region located 3’ of *lytA_Spn_* in a non-lysogenic strain. In fact, previous studies had already provided different kinds of evidence on the existence of recombination between these genes (Romero et al., 1990; Whatmore and Dowson, 1999; Morales et al., 2010). It has also been reported that *lytA_Spn_* and *plyA* (the gene encoding the main pneumolysin, a cholesterol-dependent cytolysin) form part of a pathogenicity island in *S. pneumoniae*, characterized by the presence of direct repeats (ISs), phage-related genes, and/or genes potentially encoding virulence factors and mobility proteins (Ints, transposases). These findings also suggest that the *plyA*–*lytA* island is a recombination hotspot. At least eight genomic arrangements of this island have been recognized (Morales et al., 2015). During the present study, an additional arrangement that appears to be exclusive to some NT pneumococci was found (**Figure 2B**). It is worth mentioning that a larger protein (3628 aa residues; WP_142367134) of unknown function is encoded within the *plyA*–*lytA* island in the pneumococcal strain SMRU257.

**Figure 3:**
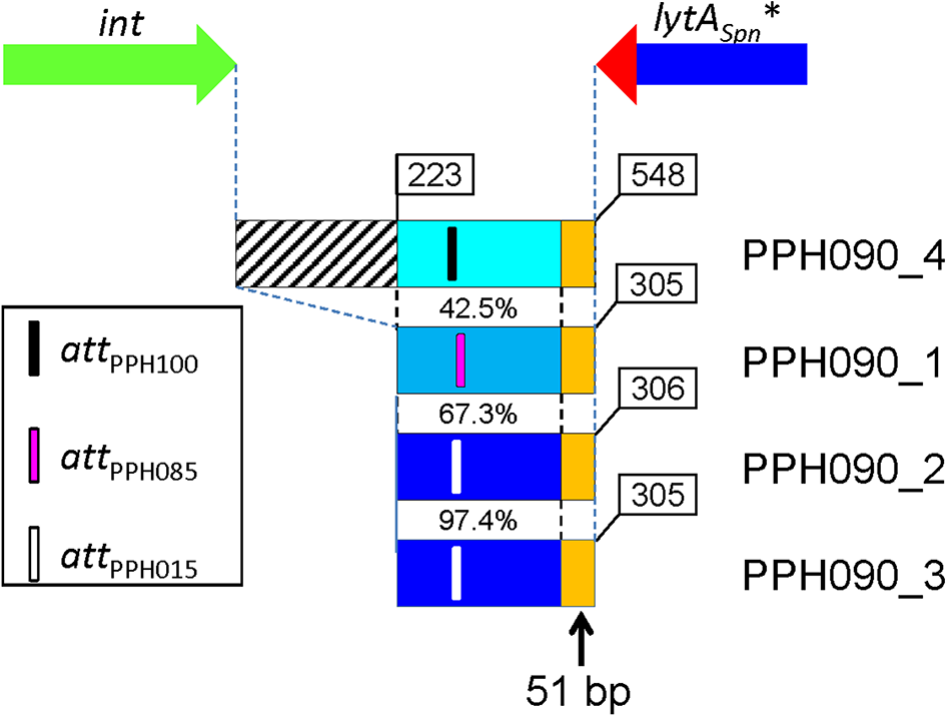
Organization of the DNA region located between the 3’ ends of the *int* and *lytA_Spn_* * genes. Genes are not drawn to scale. Four prophages of the PPH090 family were examined. The intergenic region is longer in PPH090_4 (belonging to the serine Int family) than in the other PPHs with tyrosine Ints. The percentage nucleotide identities are indicated. The approximate positions of the *attP* core sequences are shown. Two of the prophages belong to the PPH015 group and the corresponding whole regions are 97.4% identical. Orange rectangles represent the 51 bp-long perfectly conserved sequence located immediately 3’ from *lytA*_PPH_ and *lytA_Spn_* *.

The DNA region located immediately 3’ of the corresponding *lytA*_PPH_* gene of each of the four PPH090 was aligned with a 2 kb-long region located immediately downstream of the *lytA_Spn_* gene of the non-lysogenic *S. pneumoniae* D39 strain (NC_008533.2; complement [1,727,602–1,729,601; the position of the last nucleotide of *lytA_Spn_* is 1,729,602]). In agreement with the hypothesis formulated above, the four PPH090 genomes showed >96.5% nucleotide identity with the D39 genome in that region (data not shown). The PPH090 regions that run from the 3’ end of *int* to the 3’ of lytA_Spn_* after integration, were also aligned (**Figure 3**). This region was 548 bp-long in PPH090_4 (serine Int family), and shorter (305–306 bp) in prophages with tyrosine Int-coding genes. The DNA regions corresponding to the two putative PPH015 prophages (PPH090_2 and PPH090_3) were very similar (97.7%; 298 identical nucleotides in a 305 nt overlap). The presence of a 51 bp-long sequence (5’-ATAGAAAGGAAACTTTCTAAATTGTTCTTTCACCGCAGGCTTAGGCTTGCG-3’) located immediately after the termination codon of the *lytA_Spn_* * gene and found to be conserved in all the PPHs studied (indicated by orange rectangles in **Figure 3**) should be noted. Among the 126 pneumococcal strains with either complete or near complete genomes, this 51 bp-long signature was absent from the 26 non-lysogenic strains (as expected), but it was found immediately downstream of the 3’ end of the *lytA_Spn_* gene among two lysogenic strains: 4041STDY6836166 (Acc. No. NZ_LS483451; positions 1,865,445–1,865,394) and GPSC72 (Acc. No. NZ_LR216049; positions 1,745,355–1,745,304). In these strains, the 51 bp-long sequence is part of a ≈700 bp-long fragment that is ≥95% identical to another fragment located 3’ of several *lytA*_PPH_ genes, particularly those within the PPH010 group (unpublished observations). This finding indicates that those strains were once lysogenic, but that at some point, most of the prophage was lost. Interestingly, a putative transcriptional terminator is located close to the 3’ end of the *lytA*_PPH_ (and *lytA_Spn_*) genes and may function in a manner similar to that of the transcriptional terminator of the *lytA_Spn_* gene previously determined (Díaz and García, 1990) (**Figure S5**).

### Recombination Between Prophage and Bacterial *lytA* Genes Is a Source of Chromosomal Rearrangements in *S. pneumoniae*

**Figure 4:**
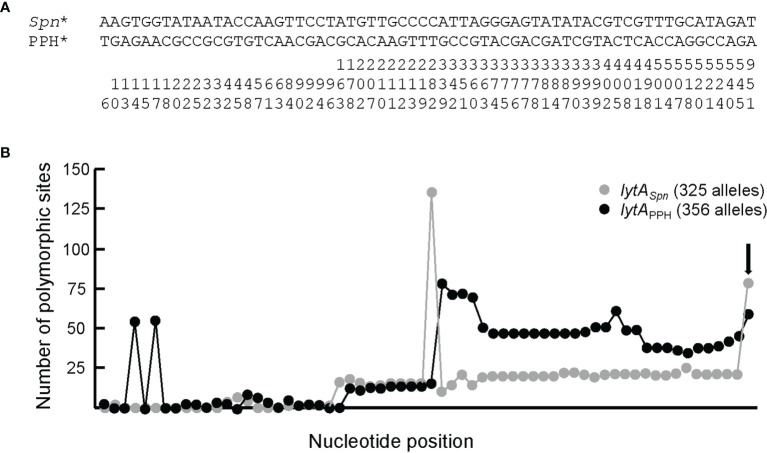
Sequence signatures of *lytA_Spn_* * and *lytA*_PPH_090_* alleles. **(A)** Bacterial and prophage *lytA* genes of strains harboring PPH090 group phages were aligned and the positions where the bacterial (on one side) and phage (on the other) genes diverged were marked. For simplicity, the highly polymorphic region characteristic of the *lytA* gene (positions 421–480) (Morales et al., 2010) was not included in the alignment. The nucleotide positions (taking 1 as the first nucleotide of the ATG initiation codon) should be read vertically. **(B)** Distribution of polymorphic sites in bacterial and prophage alleles according to the alignment shown in panel **(A)** The vertical arrow points to the data at the polymorphic position 951. The total length of the *lytA* gene is 957 bp.

**Figure 5:**
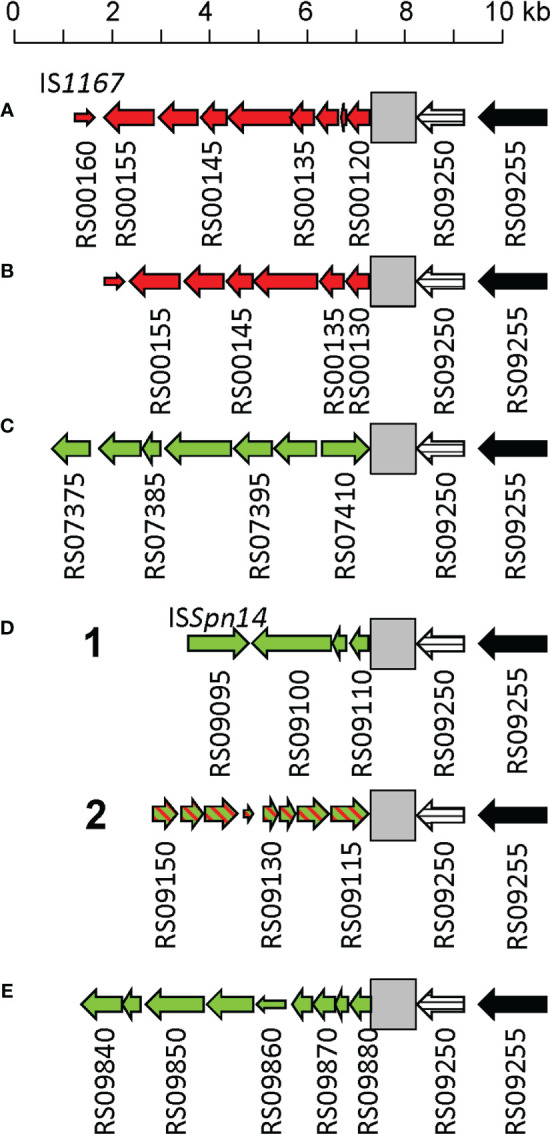
Schematic representation of several DNA rearrangements in the 3’ region of *lytA_Spn_* *. Genes are represented as arrows indicating the direction of transcription and are numbered as those of the *S. pneumoniae* D39 strain (SPD_). Narrow open arrows indicate frameshifted open reading frames. The *lytA_Spn_* gene (SPD_RS09250) is represented by a striped arrow. Panels **(A–E)** respectively show the rearrangements of genes located in the vicinity of SPD_RS00120 (coding for a nucleoside deaminase), SPD_RS00125 (*ffs* encoding the signal recognition particle sRNA), SPD_RS07415 (*whiA*), SPD_RS09110 (coding for a single-stranded DNA-binding protein), or within SPD_RS09885 (encoding ComGC, the major subunit of the competence pilus). Green and red arrows respectively indicate genes translocated or inverted with respect to their position in *S. pneumoniae* D39. In arrangement D2, an additional recombination event caused the inversion of several genes (indicated by hatched arrows). A gray square indicates the region located 3’ of *lytA_Spn_* shown in detail in **Figure 6**.

**Figure 6:**
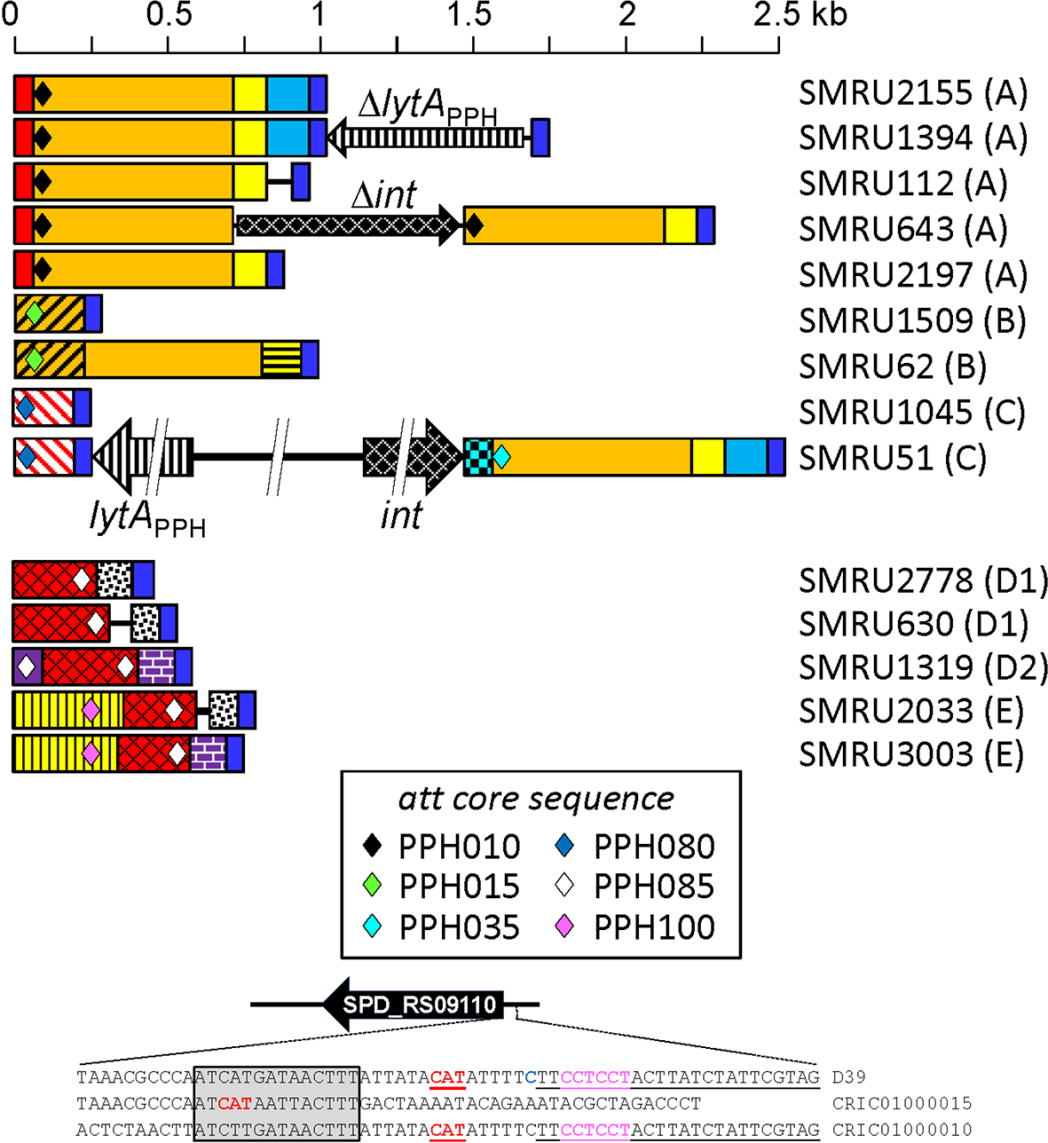
Diagram of the region located immediately downstream of the termination codon of *lytA_Spn_* * in 14 pneumococcal strains with differently rearranged genomes. The region corresponds to that indicated with a gray square in **Figure 4**. DNA regions sharing ≥95% nucleotide identity are shown with the same color and shadowing. The type of arrangement (A to E) is indicated in parentheses to the right of the name of each strain. The different core attachment sites are shown as diamonds. The deep blue rectangles represent the conserved 51 bp-long sequence mentioned in the text. The red rectangles correspond to the reverse complement of positions 24037–24121 of the *S. pneumoniae* D39 genome (between SPD_RS00120 encoding a nucleoside deaminase and SPD_RS00115 coding for an adenylosuccinate synthase). The purple rectangle corresponds to positions 1,712,485–1,712,386 (between SPD_RS09110 and SPD_RS09115 in the D39 chromosome). For additional information see **Tables S6** and **S8**. At the bottom, the nucleotide sequence surrounding the initiation codon of SPD_RS09110 in strains D39 and SMRU1319 (arrangement D2) is shown. The correct initiation codon is shown in red, bold font, and underlined. Another in-frame initiation codon is not underlined and apparently lacks a potential ribosome-binding site (RBS) (Acc. No. CRIC01000015). The predicted RBS of SPD_RS09110 is shown in pink lettering. The 14 bp-long repeat potentially responsible for a chromosomal inversion in strain SMRU1319 is inserted in a gray box. The *att* core sequence of PPH085 is underlined.

PPH090-like prophages appear to be made *via* recombination of the *lytA*_PPH_ and *lytA_Spn_* genes after the prophage is spontaneously excised from the host genome. However, recombination may also take place during the lysogenic (integrated) state of the prophage. In this instance, however, *lytA* recombination may result not in the emergence of a new PPH090 prophage but in chromosomal rearrangements. To gain insight into the recombination events that appear to occur between *lytA_Spn_* and *lytA*_PPH_, the corresponding genes from the four PPH090 reported here were aligned and the position of nucleotides conserved in lytA_Spn_* and *lytA*_PPH_* —but not in both groups— were annotated (**Figure 4A**). A search for polymorphisms at those positions was then performed in all the *lytA_Spn_* (325) and *lytA*_PPH_ (356) alleles of the dataset (**Figure 4B**). Few polymorphic positions appeared between positions 1 and 100 (approximately), but beyond there the average polymorphism reached 7 and 14% in the tested positions of *lytA_Spn_* * and *lytA*_PPH_* respectively. This suggests that recombination events (if any) are more frequent in the 3’ than the 5’ moiety of the gene. The possible significance of the notable polymorphism observed at three nucleotide positions (14 and 17 in *lytA*_PPH_, and 282 in *lytA_Spn_*) was not further analyzed, but the importance of the polymorphism at position 951 (very close to the 3’ end of *lytA* which is located at position 957) mentioned above was studied in detail. As shown in **Figure 4B**, searching among the 325 *lytA_Spn_* alleles in the resulting data showed 84 of them (154 strains) to have a ‘T’ instead of an ‘A’ nucleotide at position 951. **Table S11** summarizes a systematic analysis of this region among a subset of 51 of those 84 *lytA_Spn_* alleles (111 strains). The results indicate the existence of recombination events that produced the translocation of several DNA fragments from their original position (taking the non-lysogenic *S. pneumoniae* D39 genome as a reference) to a region located immediately downstream of the *lytA_Spn_* * gene (**Figure 5**). Five major rearrangement events were recorded and, in agreement with the genes found, the direct participation of PPH010 (SPD_RS00120), PPH015 (SPD_RS00130), PPH080 (SPD_RS07410), PPH085 (SPD_RS09110), or PPH100 (SPD_RS09880) was deduced. By comparing these arrangements with those of the different PPHs described in this study, the bacterial and phage *lytA* genes were seen to represent inverted repeats in arrangements A and B, and individual direct repeats in arrangements C, D, and E. A recombination event in cases A or B would likely produce a chromosomal inversion (**Table S11**). In addition, since the region between the *lytA* genes (the spacer) is asymmetrical around the origin of replication, a chromosomal inversion may induce changes in the relative positions of the origin and terminus of replication, as previously noted (Achaz et al., 2003). In arrangements C, D and E, a recombination event produced between repeat sequences in the same orientation on the same chromosome could result in a translocation (Hughes, 2000). A close examination of the region located immediately downstream of *lytA_Spn_* * (SPD_RS09250; depicted as gray squares in **Figure 5**) in 14 different strains revealed complex organizations of different length that contained prophage fragments or even putative full length prophages. For example, strain SMRU51 harbored a prophage equivalent to PPH035 (**Figure 6**). Of note, an additional recombination event in strain SMRU1319 (arrangement D2) produced an inversion of the genes located upstream of SPD_RS09115 and downstream of SPD_RS09110 (**Table S12**).

## Discussion

According to the data presented in this and previous studies, prophages are very common in *S. pneumoniae*: up to 90% of pneumococcal isolates may harbor temperate phages, although nearly half of them (in the present work 47%) appear to be defective and lack the genes involved in morphogenesis and lysis. These can be considered ‘defective’ (incomplete or remnant) PPHs that may be in a state of mutational decay (Casjens, 2003). It should be remembered that genome decay does not take place homogeneously. Previous reports have shown that, in incomplete prophages, there is an enrichment in *int* genes, whereas genes involved in phage lytic functions are preferentially lost (Bobay et al., 2014; Khan et al., 2020). Of note, some defective prophages are capable of excision from the bacterial chromosome (Chen et al., 2019). Sometimes, defective prophages are also designated as ‘cryptic prophages’ (Wang and Wood, 2016), although in theory this term should include only fully functional prophages that have never been induced to follow a lytic cycle (Casjens, 2003). Recently, incomplete streptococcal prophages were termed ‘satellite’ prophages (Rezaei Javan et al., 2019), although the characteristic dependence of true satellite prophages upon proteins produced by a helper prophage for packaging and progeny phage release was not demonstrated. A defective prophage (possibly a PPH005_1 relative) has been reported as capable of excising from the chromosome, but, as expected, it does not seem to enter the lytic cycle (Chen et al., 2019).

The present work fully confirms that the vast majority of full length PPHs harbor *lytA*_PPH_, a gene homologous to *lytA_Spn_*, encoding a 318-aa long NAM-amidase (*Amidase_2* family) endolysin. This clearly indicates the enhancement of phage and host fitness. A small (but significant) number of PPH endolysins (28), however, corresponded to Cpl-1-like lysozymes, and only one to a CHAP-containing enzyme. The lysozymes were the hallmark endolysins of the PPH065 and PPH105 groups. Notably, all strains harboring PPH065 were members of GPSC19 with serotype 22F (the only exception being strain 2245STDY6178828, which belongs to serotype 42). Serotype 22F is an emerging serotype (Tin Tin Htar et al., 2019) that is not included in the current 13-valent pneumococcal conjugate vaccine, although it has been incorporated into the next generation of 15- and 20-valent pneumococcal conjugate vaccines currently under evaluation (Masomian et al., 2020). All these isolates belong to clonal complex 433, which is predominant in Spain (Sempere et al., 2020). In addition, strains harboring PPH105 are of serotype 35B (sequence type 558): a single-locus variant of the PMEN Utah^35B^-24 clone, also an emerging, multidrug-resistant serotype associated with non-vaccine serotypes (Andam et al., 2017). It has been shown that pediatric isolates of serotypes 22F form better biofilms than adult isolates (Sempere et al., 2020) and that serotype 35B pneumococci are good biofilm formers (Domenech et al., 2015). Whether this phenotype is directly related to the presence of prophages, as has been proposed (Loeffler and Fischetti, 2006; Carrolo et al., 2010; Harvey et al., 2016), remains to be determined. Previous experiments have shown that the Cpl-1 endolysin is very effective in destroying *in vitro* formed biofilms either of *S. pneumoniae*, or of the type strains of *S. pseudopneumoniae* and *S. oralis* (Domenech et al., 2011). In addition, a recent study has shown that ClyJ-3 (an engineered chimera with a very short linker) shows improved thermal stability and has greater bactericidal activity against pneumococci, as well as reduced cytotoxicity, than does its parental enzyme (Yang et al., 2020). Since the Cpl-1-like lysozymes found in the present work also have linkers shorter than that of Cpl-1, detailed *in vitro* and *in vivo* studies of the newly discovered PPH lysozymes are warranted.

Using a subset of the database formed by complete or near complete pneumococcal genomes, up to 24 different *attB* sites were detected. Taking into account the capacity of some prophages to insert at different sites, the existence of 20 different PPH groups was disclosed. Various prophages integrate into genes encoding csRNAs. In *S. pneumoniae*, these non-coding sRNAs show a high degree of similarity to each other, and have been shown to affect pneumococcal physiology pleiotropically, e.g., they are involved in β-lactam resistance, and regulate natural competence development. The mechanism of action of csRNAs appears to be additive (Brantl and Brückner, 2014). Previous studies have also reported that prophage insertion into SPD_RS09885 interrupted competence development (Croucher et al., 2011; Croucher et al., 2014; Croucher et al., 2016) and it was found that having an intact *comGC* (also named *comYC*) gene was significantly associated with increased carriage duration (Lees et al., 2017).

A tandem prophage insertion (PPH080_1AB) was also found. This is usually interpreted as the integration of two phages that use the same *att* (in this case, 5’-TTATAATTCATCCGC-3’). The differences between the parental prophages in PPH080_1B involve a tandem duplication of a cluster of nine genes in PPH080_1A (marked with different colors in **Figure S2**). Further, some rare events related to PPH insertion were also noted. PPH075, PPH080_3, and PPH120 contained *attB* sequences, which would allow another phage to be integrated into their genomes, forming a prophage-in-prophage configuration and allowing the duplication of prophages possibly encoding medically or biologically important genes. To the best of our knowledge, this phenomenon has only previously been described in Shiga toxin-encoding prophages in *E. coli* (Nakamura et al., 2021).

Although first described in phage T4 in 1968 (Weiss et al., 1968), the role of tRNA genes in phages has been subject to debate (Bailly-Bechet et al., 2007). Viruses, which depend on the host protein expression machinery, have evolved various strategies to optimize translation, either by adapting their host codon usage to that of the host or encoding their own tRNAs. It has been noted that most phages contain only one or two tRNA genes, while a few may contain ≥20 (Bailly-Bechet et al., 2007). In general, temperate phages harbor fewer tRNA genes than do virulent phages. It has been suggested that tRNA acquisition may contribute to greater virulence (Bailly-Bechet et al., 2007). A more recent study of different mycobacteriophage clusters suggests that the benefits of having tRNA genes may be associated with either a better growth in their hosts (larger burst size and shorter latency) or the ability to infect more hosts (Delesalle et al., 2016). Although an increasing number of prophages are described that carry tRNA genes (Canchaya et al., 2004), their presence among *Streptococcus* prophages appears to be uncommon. In a recent analysis of 13,200 viral sequences (Morgado and Vicente, 2019), only four streptococcal phages were found to harbor tRNA genes; three were virulent [SMP from *S. suis* (Acc. No. EF116926), SP-QS1 from *S. pneumoniae* (Acc. No. HE962497), and P5652 from *Streptococcus thermophilus* (Acc. No. KY705261)], and only one was temperate [ΦARI0746 from *S. pneumoniae* (Acc. No. KT337365)].

All the VapE*_Ssu_*-like proteins were encoded by defective prophages (with the exception of PPH08_12), as previously reported (Rezaei Javan et al., 2019). Both defective and complete prophages coded for VapE*_Dno_* homologs. Experimental evidence exists that the VapE*_Ssu_* homolog of the defective prophage Javan757 (Acc. No. WP_000434359) is involved in virulence. Using a sepsis model, it was reported that mice infected with the wild-type pneumococcal strain had significantly greater blood and spleen bacterial counts than the isogenic Δ*vapE* mutant (Rezaei Javan et al., 2019). In addition, it was observed that the reduced virulence of the mutant could be explained by a significant growth delay when cultivated in serum. Further, *vapE* was upregulated only when the pneumococcal strain grew under planktonic conditions, analogous to bacteremic growth. Whether the other VapE*_Dno_* homologs described here also play a significant role in pneumococcal pathogenicity warrants further investigation.

*S. pneumoniae* does not contain an endogenous CRISPR/Cas system; this is consistent with interference with natural transformation and thereby lateral gene transfer crucial for pneumococcal host adaptation (Bikard et al., 2012) (https://crispr.i2bc.paris-saclay.fr/). Among the defense mechanisms developed by bacteria to combat phage attack, R-M systems are pervasive. Their general role is to spot foreign DNA *via* base modifications and to defend the host DNA from restriction enzymes *via* Mtase activity. Recently, however, more functions for R-M systems have been described (Vasu and Nagaraja, 2013). Their activities are due to several heterogeneous proteins classified into at least four groups. Most *S. pneumoniae* isolates express either the DpnI or DpnII R-M system, although a DpnIII system has also been reported (Eutsey et al., 2015). Interestingly, the virulent pneumophage Cp-1 cannot be restricted in *S. pneumoniae* because it does not contain the corresponding target sequence (5’-GATC-3’) in its genome (Martín et al., 1996). The virulent pneumophage Dp-1 probably defends against host-induced DNA restriction by incorporating modified bases into it (García et al., 2005). This is consistent with a more recent report showing that resistance to Dp-1 in *S. pneumoniae* results from mutations in a single gene (SPD_RS05930) coding for a type IV restriction endonuclease (Leprohon et al., 2015). It is noteworthy that type IV restriction systems differ from other types in that the Mtase and endonuclease activities are combined in a single enzyme that requires base modification to act (Loenen and Raleigh, 2014). A phase-variable type I R-M system has also been identified in various strains of *S. pneumoniae*. This system operates as an epigenetic switch that regulates gene expression, virulence, and phase variation (opaque versus transparent phenotype) in pneumococci (Li and Zhang, 2019). Moreover, the importance that phase-variable type I R-M systems have in the multifunctional defense against prophage SpSL1 infection in *S. pneumoniae* has been demonstrated (Furi et al., 2019).

The present study provides clear evidence of recombination events between pneumococcal and phage *lytA* homologs. Recombination is apparently independent of the phage Int and is facilitated by the noticeable sequence similarity between the phage and host genes (≥85% identity). Although uncommon, a similar process has been reported to occur between the *thyP3* and the *thyA* genes encoding the thymidylate synthase of the temperate bacteriophage φ3T and that of the *Bacillus subtilis* host (Tucker, 1969; Stroynowski, 1981; Stout et al., 1998; Fox et al., 1999). In this case, the nucleotide sequence identity reaches 96% (Kenny et al., 1985; Tam and Borriss, 1995). Unfortunately, whether recombination between the *B. subtilis* and the phage φ3T genes also causes genome rearrangements, as is the case of the PPH090 group, is unknown.

The present results provide a comprehensive view of the lysogenic state of phages in *S. pneumoniae*. As in most phage genomes currently under study, the majority of the PPH genes play uncharacterized roles. There is increasing evidence that although bacteriophages do not infect eukaryotic cells, they do interact with innate immune cells *via* Toll-like receptors (which appears to be particularly true for temperate bacteriophages) (Cieślik et al., 2021; Podlacha et al., 2021; Popescu et al., 2021), but the phage components involved in this are virtually unknown.

Finally, the consequences of genome rearrangements involving *lytA* genes in bacteria and phage physiology deserve to be further studied.

## Author’s Note

This work is dedicated to our mentor and friend, Concepción Ronda, who fostered the research on pneumococcal bacteriophages in our laboratory.

## Data Availability Statement

The original contributions presented in the study are included in the article/**Supplementary Material**. Further inquiries can be directed to the corresponding author.

## Authors Contributions

EG designed the study. AM-G and EG conducted the analyses and wrote the article. All authors contributed to the article and approved the submitted version.

## Conflict of Interest

The authors declare that the research was conducted in the absence of any commercial or financial relationships that could be construed as a potential conflict of interest.

## Publisher’s Note

All claims expressed in this article are solely those of the authors and do not necessarily represent those of their affiliated organizations, or those of the publisher, the editors and the reviewers. Any product that may be evaluated in this article, or claim that may be made by its manufacturer, is not guaranteed or endorsed by the publisher.

## Abbreviations

aa, amino acid(s); CHAP, a cysteine, histidine-dependent amidohydrolase/peptidase domain; csRNA, cia-dependent small RNA; GPSCs, Global Pneumococcal Sequence Clusters; Int, integrase; IS, insertion sequence; *lytA_Spn_*, gene coding for the major pneumococcal autolysin; *lytA*_PPH_, phage gene encoding a NAM-amidase of the *Amidase_2* family; NAM-amidase, *N*-acetylmuramoyl-L-alanine amidase; NCBI, National Center for Biotechnology Information; Mtase, DNA methyltransferase; NT, non-typeable; PMEN, Pneumococcal Molecular Epidemiology Network; PPH, pneumococcal prophage; RefSeq, NCBI Reference Sequence Dabatase; R-M, restriction-modification; ST, sequence type.
