# Supplementary material for: Streptococcus pneumoniae: a Plethora of Temperate Bacteriophages With a Role in Host Genome Rearrangement
Source: Front Cell Infect Microbiol. 2021 Nov 18;11:775402. doi: 10.3389/fcimb.2021.775402 (PMC8637289; doi:10.3389/fcimb.2021.775402)
Supplement: Supplementary file 1 [file DataSheet_1.zip › Figure S5.pdf]

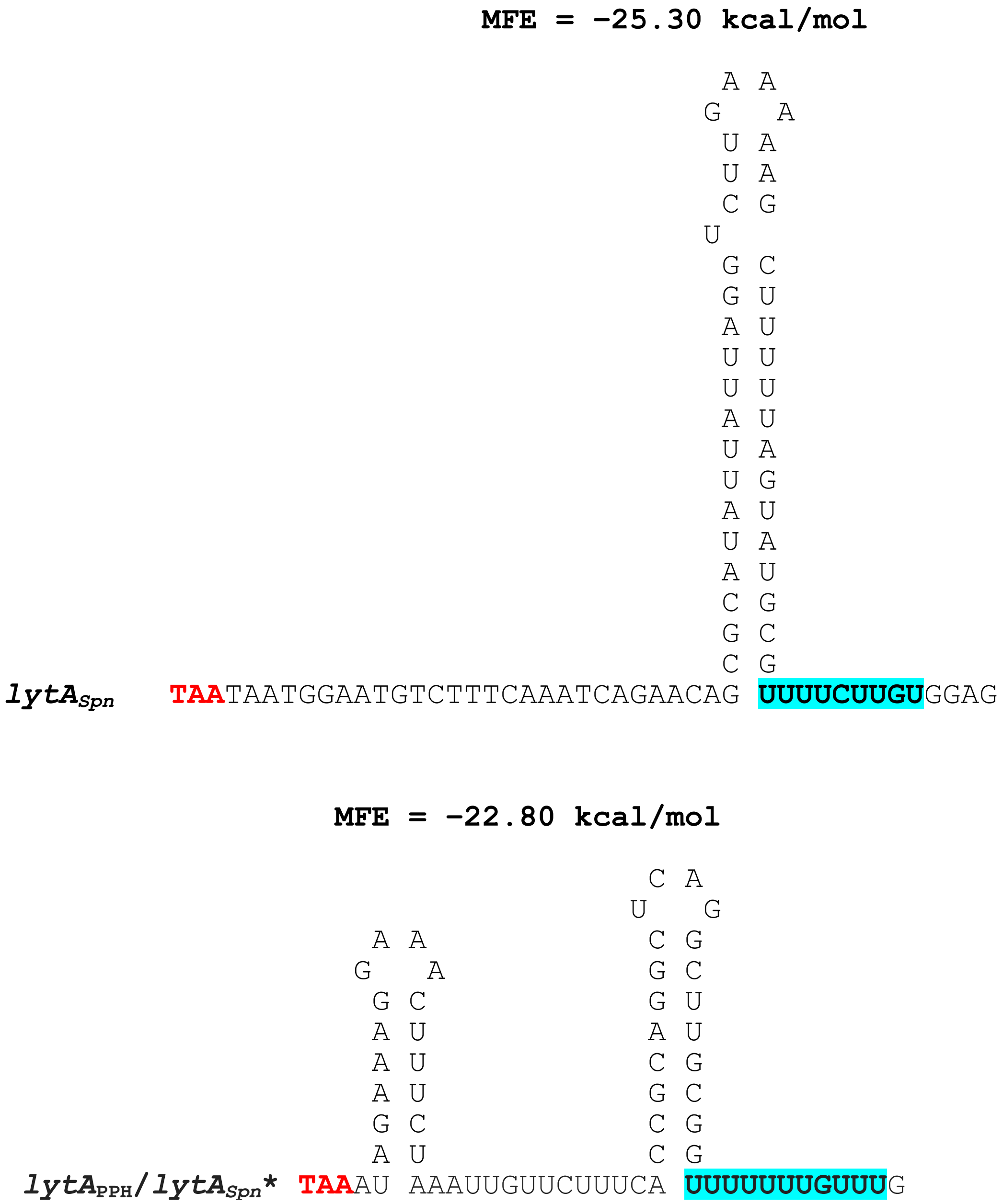
MFE = −25.30 kcal/mol
lytASpn
TAATAATGGAATGTCTTTCAAATCAGAACAG UUUUCUUGUGGAG
MFE = −22.80 kcal/mol
lytAPPH/lytASpn*
TAAAU AAAUUGUUCUUUCA UUUUUUUGUUUG

FIGURE S5
