# Supplementary material for: Streptococcus pneumoniae: a Plethora of Temperate Bacteriophages With a Role in Host Genome Rearrangement
Source: Front Cell Infect Microbiol. 2021 Nov 18;11:775402. doi: 10.3389/fcimb.2021.775402 (PMC8637289; doi:10.3389/fcimb.2021.775402)
Supplement: Supplementary file 1 [file DataSheet_1.zip › Table S7.pdf]

TABLE S7 | Sensitivity of the PHASTER program to detect prophages in our dataset.

PPH group	Dataset (Reference prophages)	PHASTER prediction (Sensitivity)[a]
005	21	12
010	35	33
015	25	22
020	4	0
025	1	0
030	25	16
035	1	1
040	1	1
045	10	0
050	1	1
055	3	3
060	2	0
065	2	2
070	11	7
075	1	1
080	21	21
085	2	2
090	4	4
095	1	1
100	5	4
105	1	1
110	1	1
115	4	2
120	1	1
125	1	0
130	1	1
TOTAL	185	137 (0.74)

[a] Sensitivity (Sn) is obtained by: (reference prophages detected/total reference prophages).
