# Supplementary material for: Streptococcus pneumoniae: a Plethora of Temperate Bacteriophages With a Role in Host Genome Rearrangement
Source: Front Cell Infect Microbiol. 2021 Nov 18;11:775402. doi: 10.3389/fcimb.2021.775402 (PMC8637289; doi:10.3389/fcimb.2021.775402)
Supplement: Supplementary file 1 [file DataSheet_1.zip › Legends to Supplemental figures.pdf]

LEGENDS TO SUPPLEMENTARY FIGURES

FIGURE S1 | Sequence alignment of the *Amidase_2* domains of four NAM-amidases. The zinc-binding residues of LysGH15 (PDB ID: 4OLS) (Gu et al., 2014) are indicated by filled blue triangles, and the catalytic residues are indicated by filled blue squares. Strictly conserved residues are written in white on a red background. Filled green triangles and filled green squares indicate the zinc-binding residues and the catalytic residues of the NAM-amidase of the pneumococcal TIGR4 strain (LytA_TIGR4; PDB ID: 4IVV) (Mellroth et al., 2014; Li et al., 2015). Other important residues for enzymatic activity are written in black on a light blue background. Other matching residues are shadowed in gray. WP_050202036 is the most common allele among the PPH endolysins studied in this study (99 occurrences in PPH080).

FIGURE S2 | Schematic representations of PPH genomes. Genes are drawn as arrows that indicate the direction of transcription. White and gray arrows correspond, respectively, to complete or interrupted bacterial genes flanking the different prophages. Within each group, the different prophages were pairwise aligned with the first one (used as query) and sequences that were ≥90% identical are shown as red rectangles with figures that correspond to 1 kb-long nucleotide sequences. When one of these rectangles in the query genome is complete in other PPH, figures are shown in white letters. Otherwise, black lettering was used. Additional similarities among other members of each group are indicated by identical shadowing. For an easier scheme interpretation, the function attributed to some gene products is also shown above the corresponding query prophage of each group. Integrase- and endolysin-coding genes are highlighted on an orange or yellow background respectively. Blue and green small rectangles indicate the positions of *attL* or *attR* respectively. Lysogenic strains are identified by their accession numbers.

FIGURE S3 | Schematic representation of diverse PblB-like phage proteins. Similar regions are indicated by identical color and shading. The percentages of sequence identity are shown at the bottom of the figure. PblB from the *S. mitis* prophage SM1 was not included in the alignments. Δ, deletion.

FIGURE S4 | Partial schematic comparison of two contigs of the NT *S. pneumoniae* strain SMRU257 with near identical PPHs with evidences of recombination between *lytA* genes. Only the DNA regions close to *lytA* are shown. A, PPH015. Note that *attL* and *attR* are 21 bp-long (5'-TTGTGTGCTCTTTTTTCGTGC-3') rather than the canonical 22 bp-long $attB_{\mathrm{PPH015}}$ (5'-TTGTGTGCTCTTTTTTTCGTGC-3'). Nucleotide sequence at the 3' end of genes $lytA_{Spn}$*, $lytA_{\mathrm{PPH}}$, and $lytA_{\mathrm{PPH}}$*. Asterisks indicate that the phage and bacterial *lytA* genes were probably hybrid because they contain sequences from both phage (red) and bacterial (blue) origins.

FIGURE S5 | Predicted secondary structure of the mRNAs from $lytA_{Spn}$ and $lytA_{\mathrm{PPH}}$/$lytA_{Spn}$* genes. The proven transcriptional terminator of the $lytA_{Spn}$ gene was previously reported (Díaz and García, 1990). The termination codons are indicated in red lettering and the stretches of U residues typical of prokaryotic transcriptional terminators are labeled in blue. The minimum free energy (MFE) of RNAs was computed using RNAfold, included in the ViennaRNA software package (http://rna.tbi.univie.ac.at/cgi-bin/RNAWebSuite/RNAfold.cgi).
