# Supplementary material for: Streptococcus pneumoniae: a Plethora of Temperate Bacteriophages With a Role in Host Genome Rearrangement
Source: Front Cell Infect Microbiol. 2021 Nov 18;11:775402. doi: 10.3389/fcimb.2021.775402 (PMC8637289; doi:10.3389/fcimb.2021.775402)
Supplement: Supplementary file 1 [file DataSheet_1.zip › Table S5.pdf]

TABLE S5 | Previously reported prophage core attachment sites (*attB*) in the *S. pneumoniae* genome.

	attB (5'→3')[a]		
Prophage	*S. pneumoniae* D39 (NC_008533.2)	*S. pneumoniae* D39V (NZ_CP027540.1)	Reference
PG1	2946 TCCCTTTTTGTGTTA 2960	2946 TCCCTTTTTGTGTTA 2960	(1)
ϕSpn_OXC/ϕSpn_3, PG2A, nameless	24016 CTTTTTCATAATAATCTCCCT 24036[b]	24016 CTTTTTCATAATAATCTCCCT 24036	(1-3)
Nameless	24694 TTGTGTGCTCTTTTTTTTCGTGC 24715[c]	24694 TTGTGTGCTCTTTTTTTTCGTGC 2471	(3)
PG2B	231378 CTTTTTCATAATAATCTCCCTTAAC 231402[d]	231834 CTTTTTCATAATAATCTCCCTTAAC 231858	(1)
MM1, VO1, MM1-1998, MM1-2000, PG3, nameless	1415148 TTATAATTCATCCGC 1415162[e]	1415604 TTATAATTCATCCGC 1415618	(1-5)
PG4	1712415 TTCCTCCTACTTATCTATTCGTA 1712437	1712871 TTCCTCCTACTTATCTATTCGTA 1712893	(1)
PG5	1841058 ACCAACATCTCCACCAA 1841074	1841514 ACCAACATCTCCACCAA 1841530	(1)

[a] The coordinates correspond to those in the genome of *S. pneumoniae* D39 (NC_008533.2) or *S. pneumoniae* D39V (NZ_CP027540.1).

[b] A 'T' and a 'C' are missing in ref. 3 (5'-CTTTTCATAATAATCTCCT-3').

[c] The sequence indicated in ref. 3 is: 5'-TTGTGTGCTCTTTTTTT(T)YGTGC-3'.

[d] The sequence indicated in ref. 3 is: 5'-CTTTTCATAATAATCTCCT-3'.

[e] The sequence indicated in ref. 3 is the antiparallel and complementary: 5'-GCSGATGAATTATAA-3'.

REFERENCES

1. Càmara, J., Cubero, M., Martín-Galiano, A. J., García, E., Grau, I., Nielsen, J. B. et al. (2018). Evolution of the β-lactam-resistant *Streptococcus pneumoniae* PMEN3 clone over a 30-year period in Barcelona, Spain. *J. Antimicrob. Chemother.* 73, 2941–2951. https://doi.org/10.1093/jac/dky305.
2. Romero, P., Croucher, N. J., Hiller, N. L., Hu, F. Z., Ehrlich, G. D., Bentley, S. D. et al. (2009). Comparative genomic analysis of ten *Streptococcus pneumoniae* temperate bacteriophages. *J. Bacteriol.* 191, 4854–4862. https://doi.org/10.1128/jb.01272-08.
3. Garriss, G., and Henriques-Normark, B. (2020) Lysogeny in *Streptococcus pneumoniae*. *Microorganisms* 8, 1546. https://doi.org/10.3390/microorganisms8101546.
4. Gindreau, E., López, R., and García, P. (2000) MM1, a temperate bacteriophage of the 23F Spanish/USA multiresistant epidemic clone of *Streptococcus pneumoniae*: structural analysis of the site-specific integration system. *J. Virol.* 74, 7803–7813. https://doi.org/10.1128/jvi.74.17.7803-7813.2000.
5. Obregón, V., García, P., López, R., and García, J. L. (2003) VO1, a temperate bacteriophage of the type 19A multiresistant epidemic 8249 strain of *Streptococcus pneumoniae*: analysis of variability of lytic and putative C5 methyltransferase genes. *Microb. Drug Resist.* 9, 7–15. https://doi.org/10.1089/107662903764736292.
