# Supplementary material for: Streptococcus pneumoniae: a Plethora of Temperate Bacteriophages With a Role in Host Genome Rearrangement
Source: Front Cell Infect Microbiol. 2021 Nov 18;11:775402. doi: 10.3389/fcimb.2021.775402 (PMC8637289; doi:10.3389/fcimb.2021.775402)
Supplement: Supplementary file 1 [file DataSheet_1.zip › Figure S4.pdf]

A (contig NZ_CKNY01000010)

B (contig NZ_CKNY01000002)

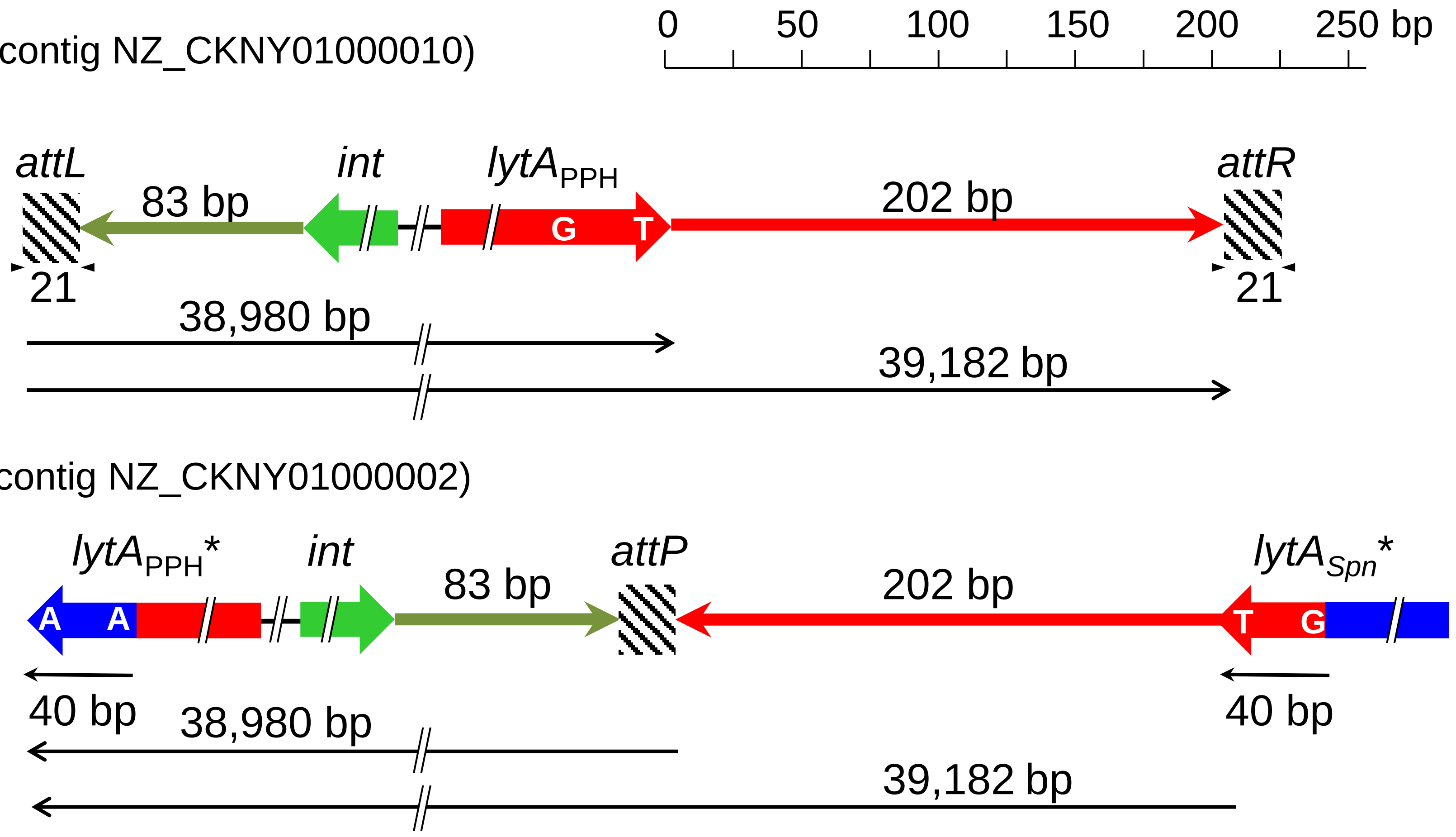

C

lytA$_{Spn}$*	901	CTGGCAGACAAGCCAGAGTTCACAGTAGAGCCAGATGGCTTGATTACAGTTAAATAA	957
lytA$_{PPH}$	901	CTGGCAGACAAGCCAGAGTTCACAGTAGAGCCAGATGGCTTGATTACAGTTAAATAA	957
lytA$_{PPH}$*	901	CTGGCAGACAAGCCAGAATTCACAGTAGAGCCAGATGGCTTGATTACAGTAAAATAA	957

FIGURE S4
