# Supplementary material for: Streptococcus pneumoniae: a Plethora of Temperate Bacteriophages With a Role in Host Genome Rearrangement
Source: Front Cell Infect Microbiol. 2021 Nov 18;11:775402. doi: 10.3389/fcimb.2021.775402 (PMC8637289; doi:10.3389/fcimb.2021.775402)
Supplement: Supplementary file 1 [file DataSheet_1.zip › Table S9 .pdf]

TABLE S9 | Pairwise alignments of $VapE_{Dno}$ and Vap_{Ssu} with PPHs-encoded proteins using TBLASTN.

	Strain (Acc. No.)	Positions[a]	PPH	Identities	Positives	$Log_{10}E$
$VapE_{Dno}$[b]						
	GPSC72 (NZ_LR216049.1)	137779–138363	020_1	63/195 (32%)	94/195 (48%)	−23
	GPSC53 (NZ_LR216061.1)	359598–360245	040	76/216 (35%)	117/216 (54%)	−37
	GPSC17 (NZ_LR216036.1)	378859–379112	050	76/216 (35%)	116/216 (53%)	−37
	GPSC7 (NZ_LR216022.1)	769754–771037	055	107/432 (25%)	173/432 (40%)	−22
	GPSC105 (NZ_LR216013.1)	2028933–2029580	075[c]	76/216 (35%)	117/216 (54%)	−37
	6A-10 (NZ_CP053210.1)	1483802–1484449	080_1A	76/216 (35%)	116/216 (53%)	−38
		1533483–1534130	080_1B	76/216 (35%)	116/216 (53%)	−38
	GPSC7 (NZ_LR216022.1)	1552486–1553133	080_2	76/216 (35%)	116/216 (53%)	−38
	11A (NZ_CP018838.1)	966067–966714	080_3	76/216 (35%)	117/216 (54%)	−37
	4041STDY6836167 (NZ_LS483448.1)	1453033–1453680	080_5	76/216 (35%)	117/216 (54%)	−37
	GPSC103 (NZ_LR216041.1)	1433464–1434111	080_6	76/216 (35%)	116/216 (53%)	−38
	NT_110_58 (NZ_CP007593.1)	1630228–1630875	080_7	76/216 (35%)	117/216 (54%)	−38
	GPSC67 (NZ_LR216034.1)	1399938–1400963	080_10	104/353 (29%)	171/353 (48%)	−37
	ND6117 (NZ_CFIR02000087.1)	5717–4431	095	109/435 (25%)	176/435 (40%)	−24
	NCTC 11898 (NZ_UHGT01000002.1)	5699–4443	110	107/423 (25%)	173/423 (40%)	−25
	GPSC78 (NZ_LR216047.1)	1966259–1967515	115_2	111/438 (25%)	169/438 (38%)	−24
Vap_{Ssu}[d]						
	Taiwan[19F]-14 (NC_012469.1)	6961–8067	005_1	126/382 (33%)	187/382 (48%)	−47
	GPSC39 (NZ_LR216025.1)	1969067–1970083	005_2	118/345 (34%)	172/345 (49%)	−44
	670-6B (NC_014498.1)	7198–8361	005_3	120/394 (30%)	183/394 (46%)	−42
	GPSC37 (NZ_LR536845.1)	2051383–2052546	005_4	120/394 (30%)	183/394 (46%)	−42
	GPSC54 (NZ_LR536833.1)	35811–36974	005_5	118/394 (30%)	184/394 (46%)	−41
	6A-10 (NZ_CP053210.1)	7256–8527	005_7	132/436 (30%)	207/436 (47%)	−47
	GPSC20 (NZ_LR216040.1)	37497–38603	005_8	126/382 (33%)	187/382 (48%)	−47
	A45 (NC_018594.1)	1898218–1899624	025	206/495 (42%)	293/495 (59%)	−115

GPSC55 (NZ_LR536843.1)	188298–189461	030_1	119/394 (30%)	183/394 (46%)	−41
SP49 (NZ_CP018136.1)	255618–256781	030_2	120/394 (30%)	184/394 (46%)	−42
GPSC25 (NZ_LR216046.1)	290689–291852	030_3	118/394 (30%)	184/394 (46%)	−41
GPSC105 (NZ_LR216013.1)	192330–193493	030_4	119/394 (30%)	183/394 (46%)	−41
	2052922–2054322	075[e]	205/493 (42%)	292/493 (59%)	−115
SP49 (NZ_CP018136.1)	1482765–1484072	080_11	166/444 (37%)	245/444 (55%)	−84

[a] Figures correspond to nucleotide positions in the genome (or contig) of the corresponding lysogenic strain.

[b] Acc. No.: WP_012030624.1 (437 aa).

[c] PPH080_5 component of PPH075.

[d] Acc. No.: WP_012027449.1 (510 aa).

[e] Defective prophage component of PPH075.
