# Supplementary material for: Streptococcus pneumoniae: a Plethora of Temperate Bacteriophages With a Role in Host Genome Rearrangement
Source: Front Cell Infect Microbiol. 2021 Nov 18;11:775402. doi: 10.3389/fcimb.2021.775402 (PMC8637289; doi:10.3389/fcimb.2021.775402)
Supplement: Supplementary file 1 [file DataSheet_1.zip › Table S8.pdf]

TABLE S8 | Predicted tRNA genes in pneumococcal prophages.

Prophage	tRNA (anticodon)	Position	References
This study			
PPH010_11	Glu (CUU)	26,373–26,444	
	Ala (CGU)	28,293–28,365	
PPH065_1	Glu (CUU)	7,103–7,175	
PPH080_11	Lys (UUU)	48,652-48,580	
	Ser (AGC)	48,988–48,898	
PPH080_12	Lys (UUU)	25,355–25,283	
	Ser (AGC)	25,691–25,601	
PPH120	Val (CAU)	73,154–73,226	
	Asp (CUG)	73,229–73,301	
	Lys (UUU)	73,326–73,398	
	Leu (GAU)	73,404–73,485	
	Thr (UGU)	73,504–73,576	
	Gly (CCG)	73,599–73,670	
	Leu (AAU)	73,678–73,763	
	Arg (GCA)	73,773–73,846	
	Pro (GGU)	73,862–73,935	
Previously described prophages			
ΦARI-0746	Ser (AGC)	18,374–18,464 /c	1
IPP8	Ser (AGC)	16,124–16,214	2, 3
IPP16	Ser (AGC)	15,754–15,844	2, 3
IPP25	Ser (AGC)	15,040–15,130	2, 3
IPP26	Ser (AGC)	15,238–15,328	2, 3
IPP27	Ser (AGC)	13,825–13,915	2, 3

REFERENCES

1. Croucher, N.J., Mostowy, R., Wymant, C., Turner, P., Bentley, S.D., and Fraser, C. (2016). Horizontal DNA transfer mechanisms of bacteria as weapons of intragenomic conflict. *PLoS Biol.* 14:e1002394. https://doi.org/10.1371/journal.pbio.1002394.
2. Brueggemann, A.B., Harrold, C.L., Javan, R.R., van Tonder, A.J., McDonnell, A.J., and Edwards, B.A. (2017). Pneumococcal prophages are diverse, but not without structure or history. *Sci. Rep.* 7:42976. https://doi.org/10.1038/srep42976.
3. Rezaei Javan, R., Ramos-Sevillano, E., Akter, A., Brown, J., and Brueggemann, A.B. (2019). Prophages and satellite prophages are widespread in Streptococcus and may play a role in pneumococcal pathogenesis. *Nat. Commun*. 10:4852. https://doi.org/10.1038/s41467-019-12825-y.
