# Supplementary figures and images for: Streptococcus pneumoniae: a Plethora of Temperate Bacteriophages With a Role in Host Genome Rearrangement
Source: Front Cell Infect Microbiol. 2021 Nov 18;11:775402. doi: 10.3389/fcimb.2021.775402 (PMC8637289; doi:10.3389/fcimb.2021.775402)

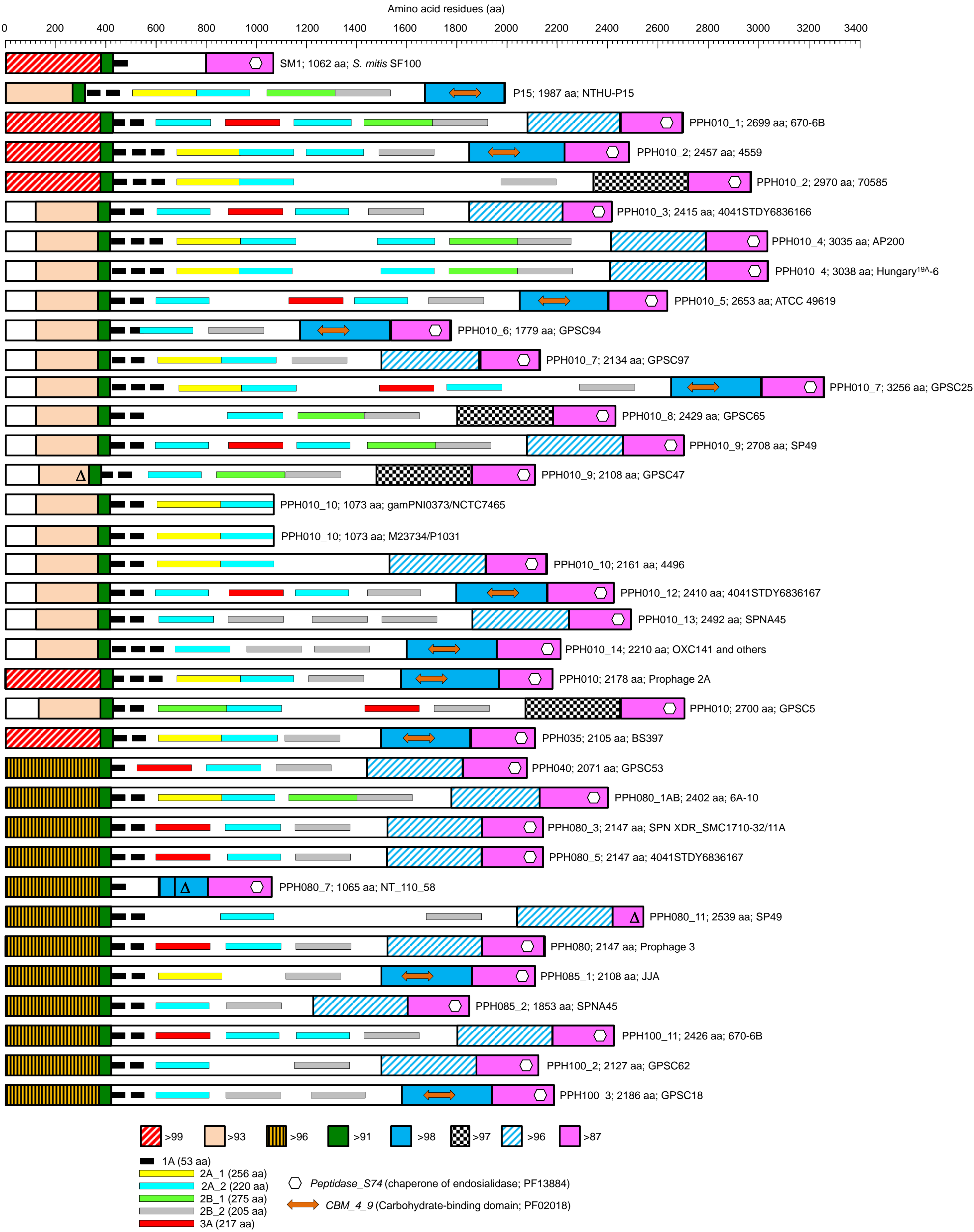

FIGURE S3

Supplement: Supplementary file 1 [file DataSheet_1.zip › Figure S3.pdf]

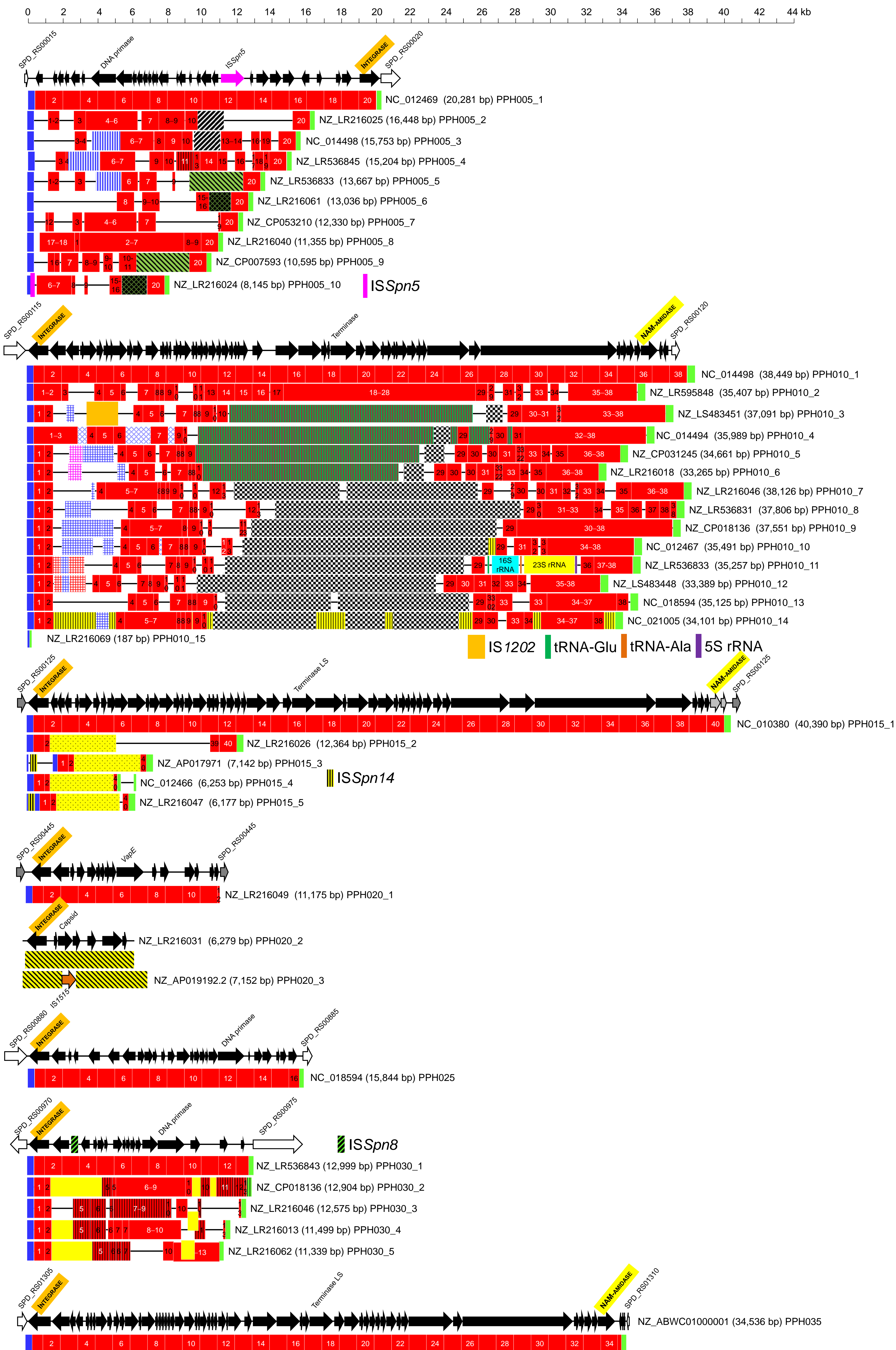

FIGURE S2

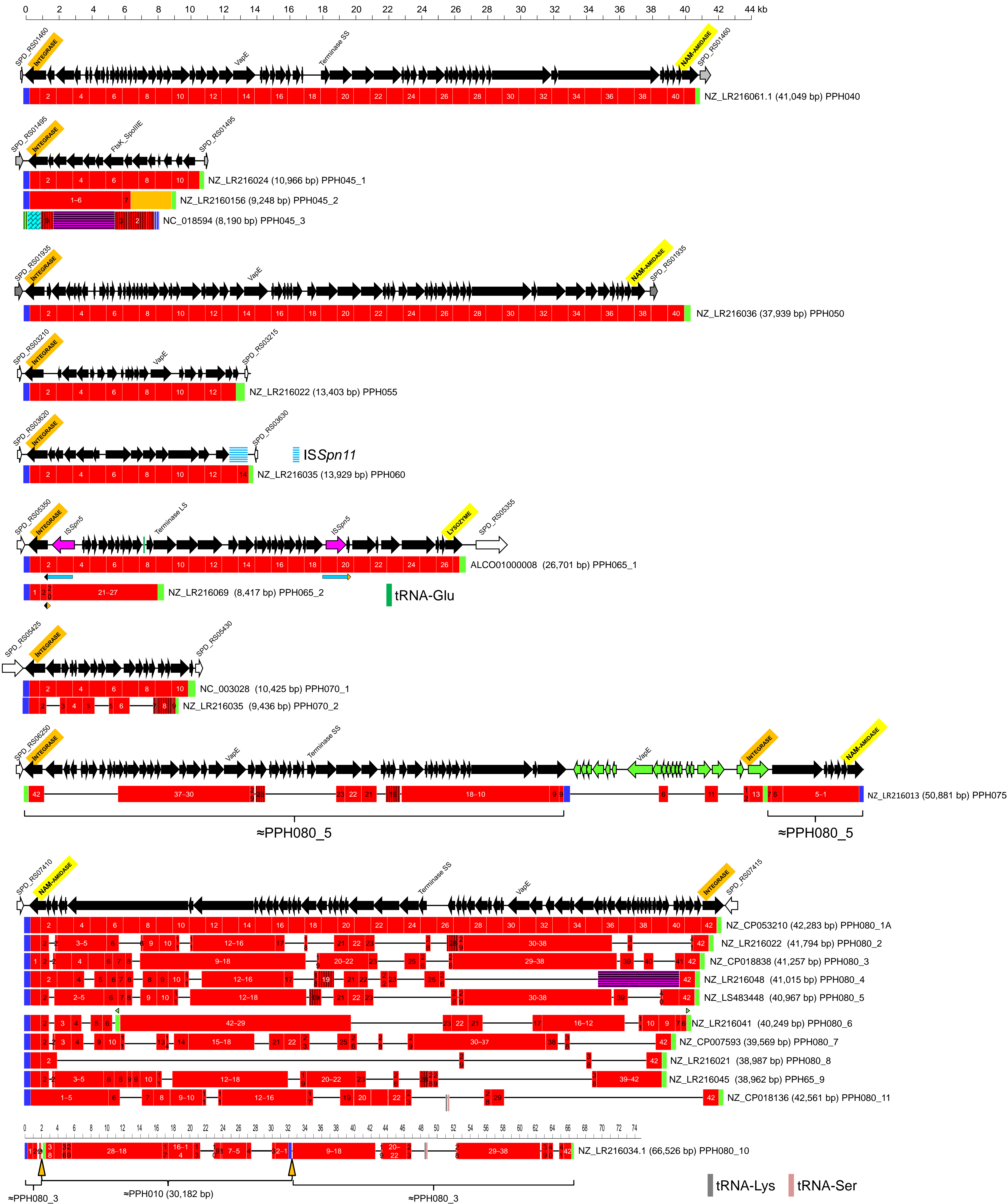

FIGURE S2

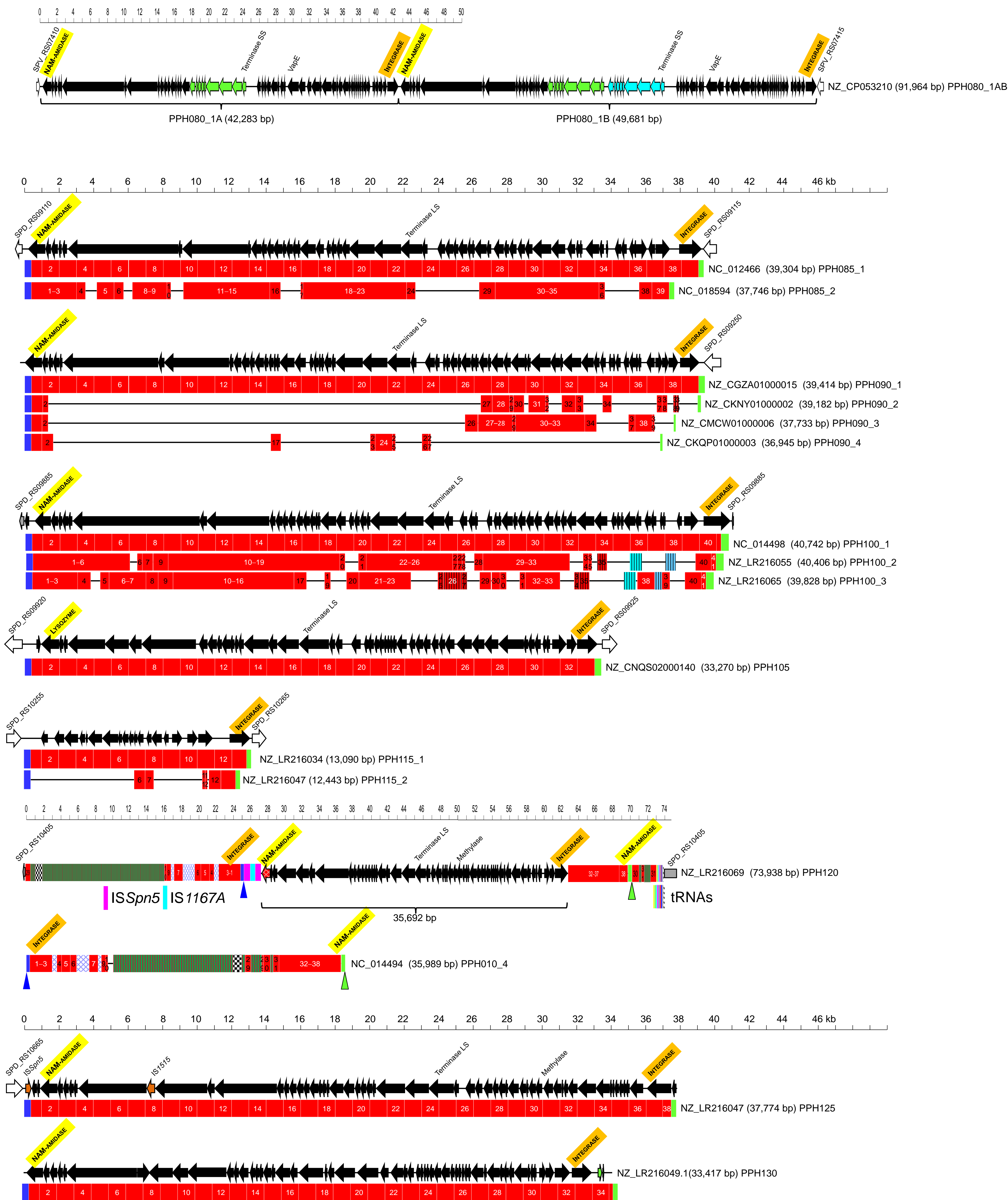

FIGURE S2

Supplement: Supplementary file 1 [file DataSheet_1.zip › Figure S2.pdf]

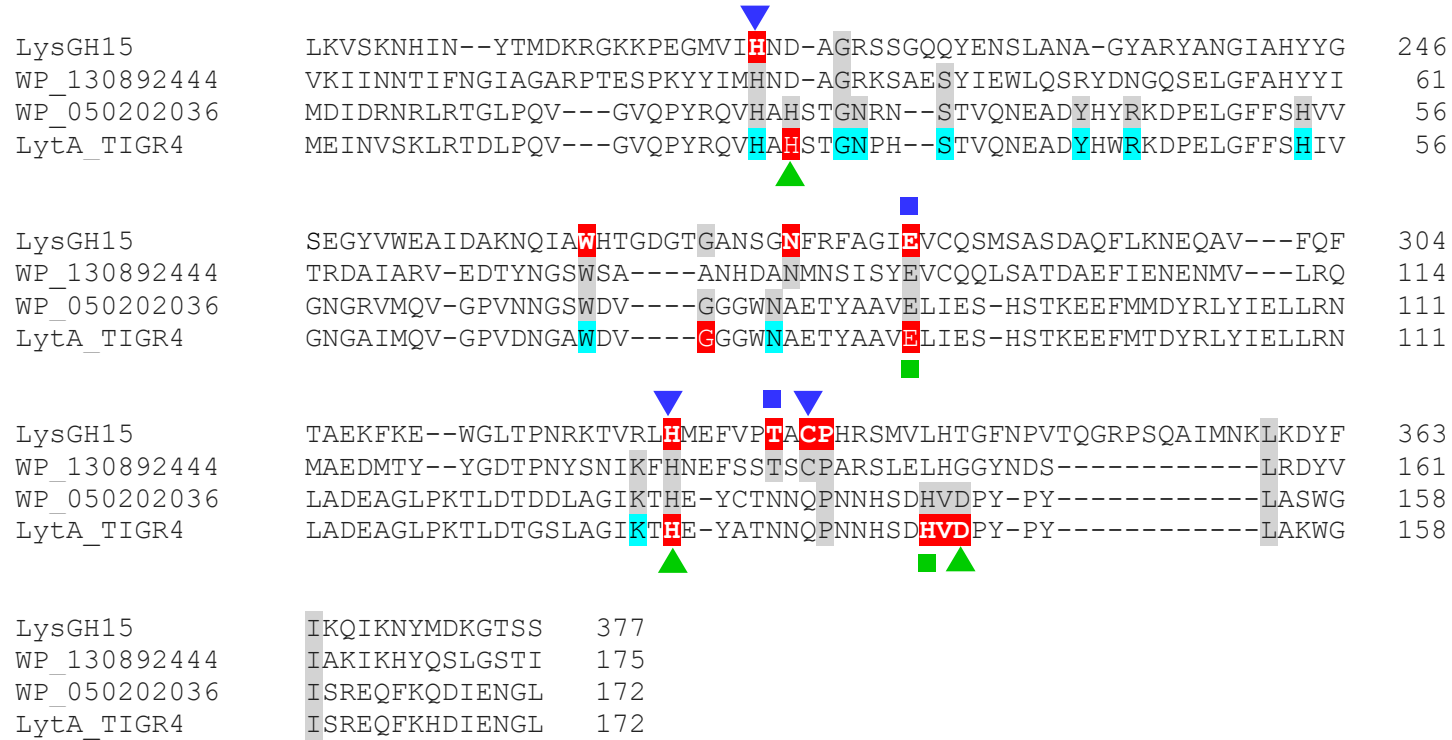

FIGURE S1

Supplement: Supplementary file 1 [file DataSheet_1.zip › Figure S1.pdf]
